# Rational design of small indolic squaraine dyes with large two-photon absorption cross section†
†Electronic supplementary information (ESI) available: Synthetic procedures and structural characterization of compounds, all the NTOs, time-resolved fluorescence spectra and the video of microscopy (Video-S1 and S2). See DOI: 10.1039/c4sc02165g



**DOI:** 10.1039/c4sc02165g

**Published:** 2014-10-07

**Authors:** Chun-Lin Sun, Qing Liao, Ting Li, Jun Li, Jian-Qiao Jiang, Zhen-Zhen Xu, Xue-Dong Wang, Rong Shen, De-Cheng Bai, Qiang Wang, Sheng-Xiang Zhang, Hong-Bing Fu, Hao-Li Zhang

**Affiliations:** a State Key Laboratory of Applied Organic Chemistry (SKLAOC) , College of Chemistry and Chemical Engineering , Lanzhou University , Lanzhou 73000 , P. R. China . Email: haoli.zhang@lzu.edu.cn; b Beijing National Laboratory for Molecular Sciences (BNLMS) Institute of Chemistry , Chinese Academy of Sciences , Beijing 100190 , P. R. China; c School of Life Sciences , Lanzhou University , Lanzhou 73000 , P. R. China; d School of Basic Medical Sciences , Lanzhou University , Lanzhou 730000 , P. R. China; e Department of Chemistry , Capital Normal University , Beijing 100048 , P. R. China

## Abstract

Small organic dyes with large two-photon absorption (TPA) cross sections (*δ*) are more desirable in many applications compared with large molecules. Herein, we proposed a facile theoretical method for the fast screening of small organic molecules as potential TPA dyes. This method is based on a theoretical analysis to the natural transition orbitals (NTOs) directly associated with the TPA transition. Experimental results on the small indolic squaraine dyes (ISD) confirmed that their TPA cross sections is strongly correlated to the delocalization degree of the NTOs of the S_2_ excited states. Aided by this simple and intuitive method, we have successfully designed and synthesized a small indolic squaraine dye (ISD) with a remarkable *δ* value above 8000 GM at 780 nm. The ISD dye also exhibits a high singlet oxygen generation quantum yield about 0.90. The rationally designed TPA dye was successfully applied in both two-photon excited fluorescence cell imaging and *in vivo* cerebrovascular blood fluid tracing.

## Abstract 2

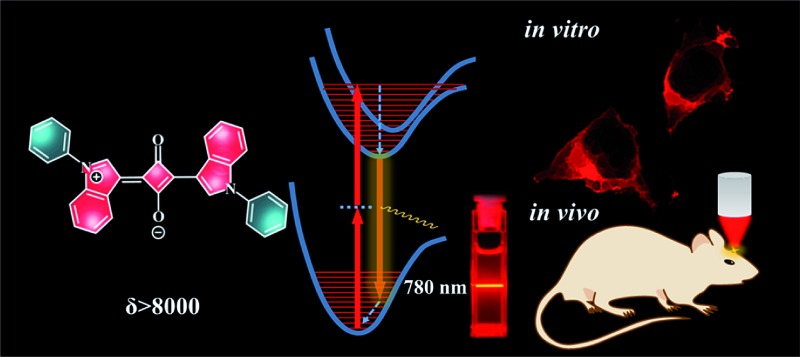
Assisted by theoretical analysis, we designed a small indolic squaraine with *δ* > 8000 GM at 780 nm, which is ideal for both *in vitro* and *in vivo* bio-imaging applications.

## Introduction

Organic materials exhibiting two-photon absorption (TPA)^1^ activity are of interest for many applications, including optical pulse suppression,^2^ two-photon fluorescence microscopy,^3^ photodynamic therapy,^4^ 3D fabrication,^5^ and 3D optical data storage.^6^ However, two-photon absorption and excitation of one molecule are intrinsically much less efficient than a one-photon process, and the occurrence probability of TPA, which is reflected by the parameter of TPA cross-section (*δ*), is generally small for most organic molecules. Therefore, the design and discovery of new TPA molecules with large *δ* values are of great importance.

Previous design of TPA dyes has generally been based on using extended π-conjugated frameworks with enforced coplanarity and addition of donor and acceptor groups.^7^ By using these strategies, most of the reported TPA dyes with high *δ* consist of large π-systems and various donor–acceptor units.^8^ However, large conjugated molecules are not always appreciated in practice because they may meet difficulties in synthesis, purification and solubility.^9^ Importantly, large conjugated chromophores tend to have small S_1_–S_0_ energy gaps, and rapid S_1_–S_0_ nonradiative internal conversion, resulting in low-energy short-lived excited states, and low quantum yield, which are undesirable for fluorescence or photochemical activations and photodynamic therapy (PDT).^7a,10^ For applications such as TPA imaging, photoinitiator and optical limiting, ideal TPA dyes should exhibit peak absorption matching the commercial laser source.^11^ Only in a few special applications, such as TPA at telecommunication wavelengths, a narrow optical gap is preferred.^12^ Therefore, maximizing the *δ* is not the only consideration when designing a TPA dye. Besides, it is often useful to pack the maximum effect into the smallest possible chromosphere. Anderson has proposed that *δ*/*N*_e_ (where *N*_e_ is the number of π electrons) can be used as a useful parameter to evaluate how efficiently the π-conjugated system contributes to the *δ*.^7*a*^ To date, there has been very few systems achieving *δ*/*N*_e_ > 200 GM, and these reported molecules tend to be either too complicated for synthesis^13^ or have too low energy gap.^8a,14^ In TPA microscopy, small TPA dyes that have peak TPA absorption in the 700–900 nm window with high *δ* and are most favorable,^15^ while an ideal TPA dye should absorb at the shortest possible wavelengths to enable the best possible resolution.^16^

Developing theoretical methods to guide the design of smaller but more efficient TPA dyes is an attractive idea.^8b,16^ Because the TPA process is a third-order nonlinear phenomenon and *δ* is related to the imaginary part of the third nonlinear polarization, theoretical calculation of *δ* is very complicated. Brédas *et al.* have studied the relationship of *δ* with single photon absorption transition dipole moment using semi-empirical AM1 and INDO calculations.^17^ Full *ab initio* calculation of the third nonlinear polarization has been studied by response theory, including the sum-over-states technique^18^ and the finite field method.^19^ Unfortunately, even with the state-of-the-art calculation tools, the theoretically predicted *δ* often showed large discrepancy with the experimental results, in some cases, by orders of magnitude.^7b,20^ Another problem with these complicated theoretical methods is that the structure–property relationship is hidden by the complex wave function calculation, and can hardly provide any intuitive information for molecular design. To date, new TPA materials are typically discovered experimentally, with theoretical characterization providing mostly *post facto* justification.^21^ Therefore, an easy-to-use theoretical method that can guide the practicable design of TPA materials is critically needed.

In this work, we report a simple theory-assisted method for the fast screening of organic molecules as potential TPA dyes. This method is based on morphology-analysis of the final excited state of the TPA process, which provides qualitative information that is useful for both pre-synthesis predication and *post facto* analysis. By applying this method on a series of indolic squaraine dye (ISD), we have successfully obtained a small ISD molecule with an exceptionally large *δ* value. The peak TPA absorption of the obtained dye is around 780 nm, which matches well with the laser source of the TPA microscope. The newly designed TPA dye shows potential in both *in vitro* and *in vivo* bio-imaging applications.

## Results and discussion

Squaraine is a relatively old class of organic dye, which has recently received a huge resurgence of interest due to their potential usefulness in a large number of technologically relevant fields.^22^ The study of squaraines as possible TPA dyes have been conducted by many groups,^4a,8a,16,23^ which suggest that the TPA performance of squaraines can be enhanced by extension of its π-system and addition of donor groups or attachment of organometallic units.^8a,24^ However, small squaraines with high *δ* were rarely reported. ISD is selected as the model structure in this work due to the ease of both synthesis and structural modification. The best small TPA dye based-on ISD framework was reported by Pagani, which has the largest *δ* up to 450 GM.^16,25^

**Fig. 1:**
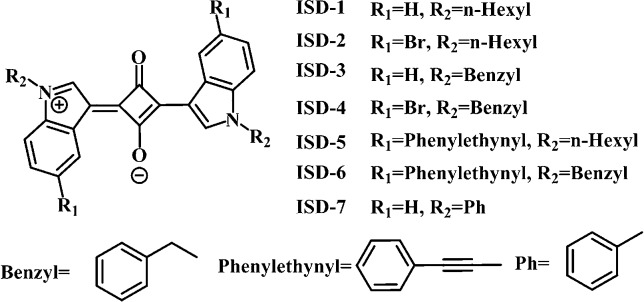
Structures and numbering scheme for the ISD compounds studied in this paper.

To unveil the structure–property relation, we have designed seven small ISD molecules, as shown in Fig. 1. Two positions were readily used in structural modification, *i.e.*, the 5-position of benzene ring and the *N*-position of the pyrrole ring. The seven molecules can be classified into two groups. From ISD-1 to ISD-4, the maximum number of π electron in conjugation is maintained at 22. The Br, *n*-hexyl and benzyl substitutions to the molecules are designed to study the effects of heavy atoms, non-conjugated alkyl and aromatic substitutions, respectively. ISD-1 is very similar to that reported in previous literature,^25^ and is used as a reference. From ISD-5 to ISD-7, the π system is expanded by directly attaching aromatic structures to the indole part (Table 1). Some other designed molecules are presented in the ESI† (part 6).

**Table 1:** Spectroscopic parameters of the ISD molecules and TPA cross sections

	ISD-1	ISD-2	ISD-3	ISD-4	ISD-5	ISD-6	ISD-7
*N*_e_ ^*a*^	22 (22)	22 (22)	22 (34)	22 (34)	38 (42)	38 (54)	22 (34)
*λ*_abs_ ^*b*^	560	561	562	561	561	578	575
*λ*_em_ ^*c*^	580	581	582	582	582	598	600
*τ* ^*d*^	1.58	1.65	1.53	1.57	1.64	2.67	1.47
*Φ* ^*e*^	0.701	0.499	0.505	0.301	0.676	0.580	0.288
*k*_r_ ^*f*^	0.444	0.302	0.330	0.192	0.412	0.218	0.196
*k*_nr_ ^*g*^	0.189	0.304	0.324	0.445	0.198	0.157	0.484
*δ*_max_ ^*h*^	330	1260	730	701	712	645	8019
*Φ*_Δ_ ^*i*^	0.30	0.62	0.50	0.77	0.70	0.64	0.90
*δ*/*N*_e_ ^*j*^	15 (15)	57 (57)	33 (21)	32 (21)	19 (17)	17 (12)	365 (236)
*δΦ*_Δ_ ^*k*^	99.7	785.8	365.1	543.0	501.9	411.4	7217.1

^*a*^ *N*_e_ are the maximum number of conjugated π electrons in the planar part with the total number of π electrons in the molecules given in parentheses.

^*b*^ *λ*_abs_ (nm) is the maximum absorption wavelength.

^*c*^ *λ*_em_ (nm) is the maximum emission wavelength.

^*d*^ *τ* (ns) is the fluorescence lifetime.

^*e*^ *Φ* is the fluorescence quantum yield.

^*f*^ *k*_r_ (ns^–1^) is the rate constant for radiative deactivation from S_1_ to S_0_.

^*g*^ *k*_nr_ (ns^–1^) is the rate constant for non-radiative deactivation.

^*h*^ *δ* (GM) is the TPA cross section.

^*i*^ *Φ*_Δ_ is the singlet oxygen quantum yield.

^*j*^ *δ*/*N*_e_ is *δ*_max_ normalized by the π electron number.

^*k*^ *δΦ*_Δ_ presents the TPA singlet oxygen generation capability.

**Fig. 2:**
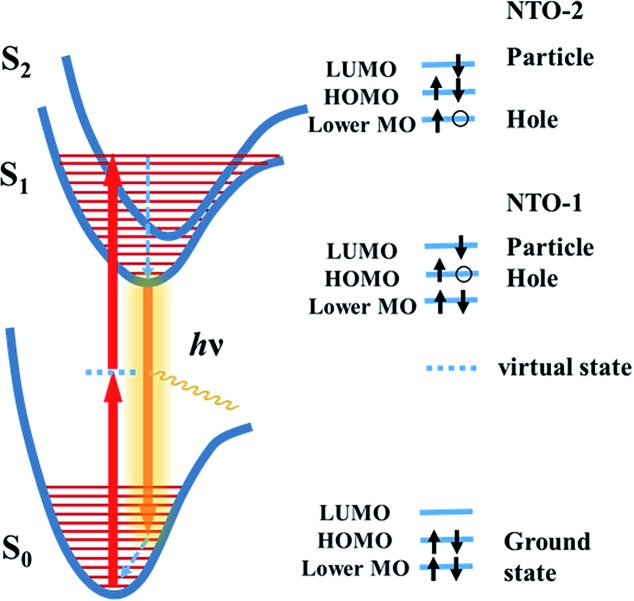
Energy level diagrams for the essential states for centrosymmetric chromophores in the TPA process and schematic diagram for the electron transition between energy levels. Lower MO represents HOMO-1 orbital or other lower orbital.

Previous studies have shown that using an extended π-conjugated framework and donor–acceptor groups are likely to enhance the *δ*.^7^ Meanwhile, it is important to be aware that the extended conjugation structure or added groups need to participate the TPA process in order to contribute to the *δ*. In a TPA process, the ground state molecule simultaneously absorbs two photons to be excited to a excited state through a virtual state (Fig. 2). Both the virtual state and the final state should have strong influence on the *δ*. Accurate description of TPA excited state need to include electron-correlation effects known as configuration interaction (CI)^26^ and thus requires complex and time-consuming calculations. Moreover, a precise calculation of the virtual state is difficult to achieve at the moment. To simplify the calculation, we focused on the final excited state of the TPA process. The electronic properties of the excited state can be derived by the calculation of natural transition orbitals (NTOs),^27^ which can be used to evaluate the participation or influence of a certain structural unit in the molecule. It is known that a high degree of delocalization of the excited states may enlarge the oscillator strength of a particular absorption and increase the corresponding transition moment.^7*a*^ In the TPA process, a highly delocalized final excited state is more likely to give a large *δ* than a localized one. Herein, we demonstrate that the visualization of the NTOs not only can help to understand the excited state associated to the TPA process but also help to screen TPA materials with high *δ*.

It is known that the TPA process depends on the symmetry of the chromophore, and the final state is different for noncentrosymmetric or centrosymmetric chromophores.^7*a*^ For dipolar noncentrosymmetric chromophores, their one- and two-photon transitions are the same, which implies that the maximum TPA transition energy is half of the energy needed in the one-photon counterpart. However, the one and two photon transitions are different for the symmetric quadrupolar chromophores, in which the TPA transition is from the ground state (S_0_, ^1^A_g_) to the lowest excited state with A_g_ symmetry (S_2_, ^2^A_g_), so that the S_2_ state is the final state in TPA process.^7^ Because the ISD compounds designed in this work are all centrosymmetric, we studied their NTOs of the S_2_ states.

**Fig. 3:**
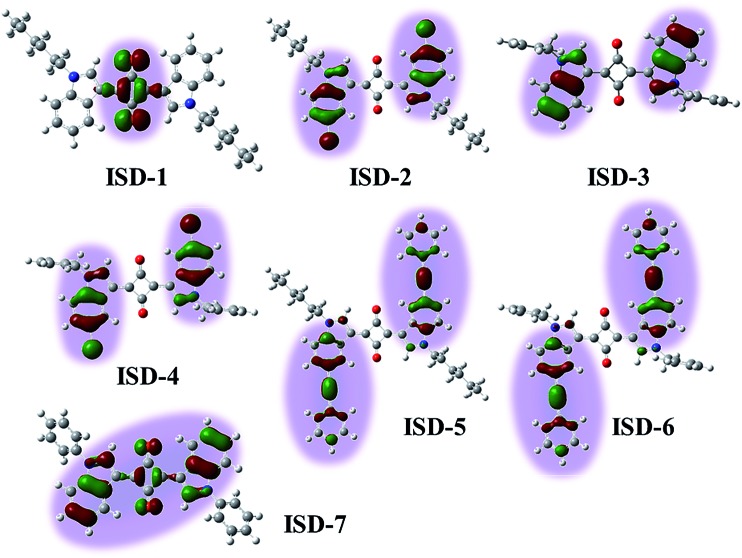
The natural transition orbitals for the “hole” in the second excited state (S_2_) of seven ISD molecules.

Fig. 2 illustrates the electron transition of centrosymmetric chromophores in a TPA process. As illustrated in Fig. 2, the final TPA excited state (S_2_ state) of the ISD molecules can be described by the particle–hole pair of the NTO-2. To study the excited states, TD-DFT calculations were conducted using the Gaussian 09 package.^28^ The detailed simulation results are collected in the ESI (Fig. S1†). Structural optimization suggests that the indolic squaraine structure, including the four-membered ring and two indolic units, has a coplanar conformation, which is ideal for an extended excited state. The planarity of ISD-5 and ISD-6 extends to the phenylethynyl groups. The NTOs of S_1_ and S_2_ were studied to visualize the excited state in the form of “electron” and “hole” distributions. We paid particular attention to the delocalization of the NTOs of S_2_, as S_2_ is the final state after absorbing two photons. The calculated “holes” of S_2_ states of the representative ISDs are shown in Fig. 3, while the other NTOs are provided in Fig. S2.†

Fig. 3 reveals that the excited states are distinctly different for the ISD molecules, despite their structural similarity. Importantly, not all parts of the conjugated structures take part in the TPA final states. For ISD-1, which is the alkyl substituted ISD, the “hole” of the NTO-2 is mainly restricted to the four-membered squaraine ring, and the benzene rings do not contribute to the excited state. When the hexyl units are replaced by the benzyl groups (such as ISD-3), the situation is very different. The excited sate of ISD-3 is mainly localized on the two indole rings. However, the four-membered ring does not participate in the excited state, so that the NTO is divided into two separated parts. The Br atoms on the indole rings are involved in the NTO-2 but they do not change the NTO morphology very much, and the NTOs of ISD-2 and ISD-4 are very similar to that of ISD-3. Expanding the π system was achieved by attaching phenylethynyl moieties to the 5-position of the indole benzene (ISD-5 and ISD-6). Seen from the “hole” of S_2_, the orbital of the excited state indeed extends to contain both the indole and the phenylethynyl units. However, the NTOs are still separated by the four-member ring, which gives limited delocalization of the TPA excited state. The most different NTO is observed on ISD-7, which has a benzene group attached on the *N*-position of each indole ring. It is very interesting that the “hole” of ISD-7 extended to cover both the squaraine core and the two indole units, though the two benzene rings attached on the N positions are not involved in the S_2_ state due to non-planar conformation.

As mentioned above, the S_2_ state is the final excited state of the TPA process. A reasonable presumption is that a high degree of delocalization of the final excited state is beneficial for the corresponding absorption process. The variations of NTO of different molecules are associated to their energy levels and orbital symmetry, and cannot be simply predicted from their molecular structures. Our NTO analysis helps to visualize the excited state and hence provides useful information to qualitatively predict their *δ* values. For compound ISD-1, its NTO of the S_2_ state is very small and locates only in the four-membered ring of the squaraine core. Therefore, it is not surprising that this type of molecules has relatively small *δ*, as reported in previous studies.^25^ In the case of ISD-2 to ISD-6, their NTO of the excited states are larger than that of the ISD-1, so that larger *δ* values are expected. However, their NTOs are separated into two parts, which is not the most ideal situation for TPA process. In contrast to all the other molecules, the calculation shows that the ISD-7 have the most delocalized excited state, and hence is expected to have the largest TPA cross section.

**Fig. 4:**
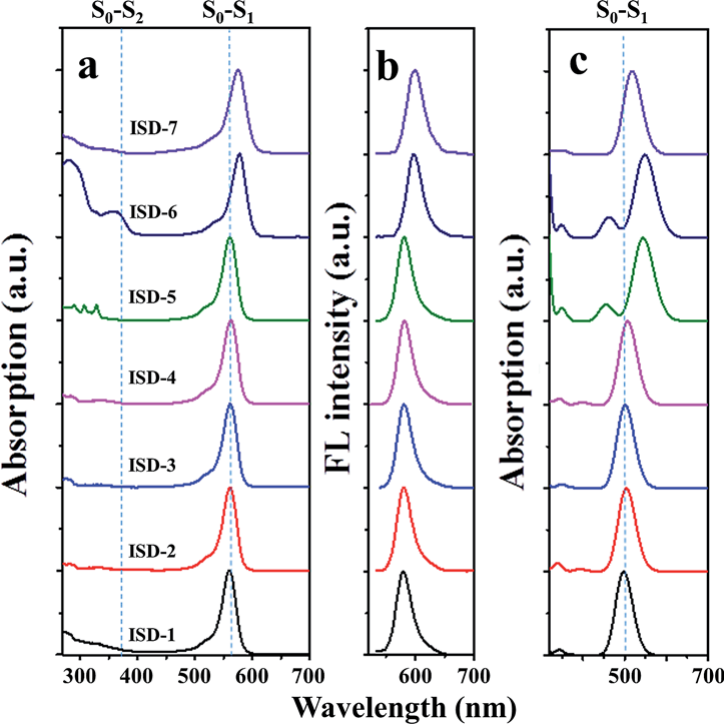
(a) Steady-state one-photon absorption and (b) fluorescence of ISD molecules in THF, and (c) the simulated absorption spectra.

To test the above hypothesis, the compounds ISD-1 to ISD-7 were synthesized and their absorption and emission properties have been characterized. The experimental one-photon absorption and emission spectra of the seven ISD molecules in diluted THF solution are shown in Fig. 4a and b, respectively; while the key parameters are summarized in Table 1. The one-photon absorption spectra have also been simulated by time-dependent density functional theory (TD-DFT) methods (Fig. 4c), which helps to give precise assignments to the absorption peaks.

The one-photon absorption spectrum of the ISD-1 shows a typical strong and sharp absorption band around 560 nm, corresponding to the S_0_–S_1_ transition. The absorption bands of ISD-2 to ISD-4 are nearly unaffected by the substitution. The main absorption bands of ISD-6 and ISD-7 show bathochromic-shifts for 18 and 15 nm, respectively. Experimental results are generally consistent with the calculated results, although certain deviations exist, which is common in TD-DFT calculation.^29^ For instance, the transition energy of S_0_–S_1_ is underestimated for ISD-5 and ISD-6 because of the presence of the C–C triple bond.^30^

The one-photon absorption spectra also indicate that the S_0_–S_2_ transition intensity (shown in the Fig. 4a) is much weaker than that of the S_0_–S_1_ transition. From the calculated results, the oscillator strengths of the S_0_–S_2_ transitions are all zero for the seven ISDs, which can be attributed to the symmetry forbidden one-photon absorption process. In practice, the S_0_–S_2_ transition intensity comes from the vibronic coupling (also called Herzberg–Teller effect) by which the symmetry-forbidden rule is broken.^31^ The selection rule is reversed between two-photon and one-photon absorption processes in general to centrosymmetric chromophores,^7^ and the S_0_–S_2_ transitions are in favor of the TPA process for our ISDs. The emission spectra of ISD-1 to ISD-5 are nearly the same, while the emissions of ISD-6 and ISD-7 are slightly red-shifted, consistent with their absorption spectra (Fig. 4b). Fig. S2† shows that the colors of ISD-1 to 7 are very similar with each other under the one-photon absorption condition, and are almost the same in their fluorescence emission. The similar color indicates very small differences exist in the one-photon absorption processes among these seven ISDs.

The fluorescent quantum yields (*Φ*) of these seven ISDs were measured and compared in Table 1. The alkyl substituted ISD-1 gives the highest *Φ* of 0.701, while introduction of all the other substituent groups tends to reduce the *Φ*. The *Φ* of the Br-containing molecules (ISD-2 and ISD-4) were suppressed compared with their non-Br counterparts (ISD-1 and ISD-3) due to the heavy-atom effect,^32^ which results in reduced radiative rate constant of *k*_r_ and concomitant enhanced non-radiative deactivation rate of *k*_nr_, as derived from the time-resolved fluorescence spectra (Fig. S3†). The *Φ* values of benzyl-containing ISDs (ISD-3, ISD-4 and ISD-6) were lower compared with the corresponding non-benzyl-containing molecules (ISD-1, ISD-2 and ISD-5). The *Φ* reduction also occurs upon phenylethynyl substitutions. The benzene modification at the *N*-position brings about the smallest *Φ* for ISD-7, which is only 0.288. The *Φ* suppression after attaching different aromatic groups probably arises from the intra-molecular rotation of the substituent groups, which provides additional channels for non-radiative de-excitation.^33^

**Fig. 5:**
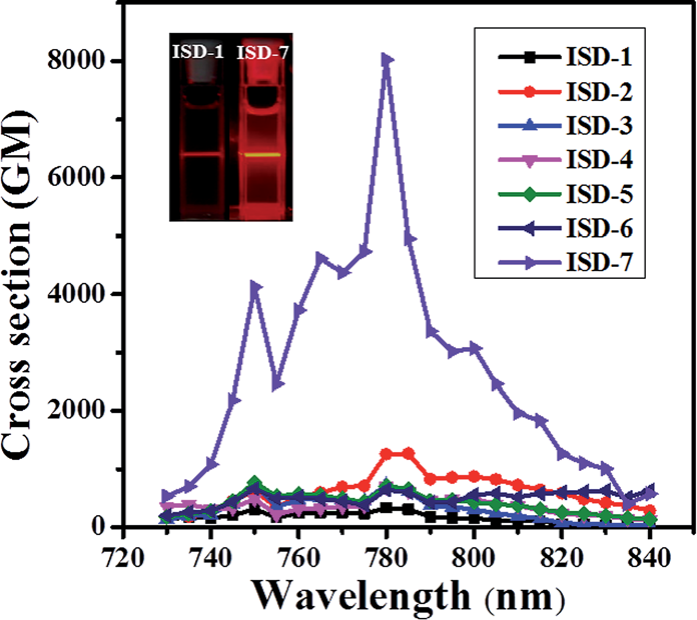
(a) TPA cross-section of ISDs in THF solutions. The inset shows the corresponding TPA fluorescence photographs of the ISD-1 (left) solution and the ISD-7 (right) solution under 780 nm illumination.

The TPA spectra between 730 and 840 nm are shown in Fig. 5, and the maximum TPA *δ* is shown in Table 1. The maximum TPA absorption occurs around 780 nm for all the studied molecules, corresponding well with the two-photon absorption for the S_0_–S_2_ transition. The TPA *δ* of ISD-1 was measured to be 330 GM under our condition, which is in good agreement with previously reported ISD molecules with similar structure (450 GM).^25^ For the substituted molecules from ISD-2 to ISD-6, the *δ* values are in the range of 600–1200 GM, much larger than that of the model molecule ISD-1. This result is consistent with our theoretical calculation results, where ISD-2 to ISD-6 have larger NTOs of the S_2_ states compared with that of ISD-1. It is very interesting that the TPA *δ* of ISD-2 is about 1200, three times larger than that of ISD-1. As discussed before, the halogenation effect to the photoluminescence process is generally very complicated.^29b,34^ From the NTOs of ISD-2, such TPA *δ* enhancement in ISD-2 can be attributed to the participation of the Br-atom in the TPA process.

It was discovered that the *δ* of ISD-7 is exceptionally high, reaching 8019 GM, which is more than 20 times higher than that of ISD-1. Indeed, two-photon excitation fluorescence shows that the ISD-7 is significantly brighter than ISD-1 under the same condition (inset of Fig. 5). Such a high *δ* value is in excellent agreement with our theoretical calculation that ISD-7 has the most extended NTO of the S_2_ states. It is noted that the *δ* value of ISD-7 measured under our condition is about 17 times higher than the largest *δ* value ever reported for indolic squaraine derivatives.^16,25^ If we only consider the π electrons in the planar part, the *δ*/*N*_e_ is as high as 365 GM, which is almost the highest for all the known organic TPA dyes with maximum absorption below 800 nm.^7a,13^ The successful design of ISD-7 strongly suggests that the proposed NTO morphology analysis method is an efficient method for the rational design of organic dyes with large TPA cross sections.

The above experimental results confirmed that extension of the π-system is not necessarily a useful way to increase *δ*.^7*a*^ For instance, the molecules with the largest number of π electrons in this work, *i.e.* ISD-5 and ISD-6, did not show very high *δ* values, indicating that the expansion of the π system does not necessarily enlarge *δ*. The π-expansion strategy is only effective when the additional π structures participate in the TPA process. We also found that the non-planar substitution, such as the phenyl groups in ISD-7, may facilitate the *δ* enlargement by extending the NTOs associated with TPA absorption. In addition, we note that one should be cautious to directly use the linear optical properties, such as one-photon absorption, fluorescence spectra, fluorescence lifetime and quantum yield, to design TPA dyes.

**Fig. 6:**
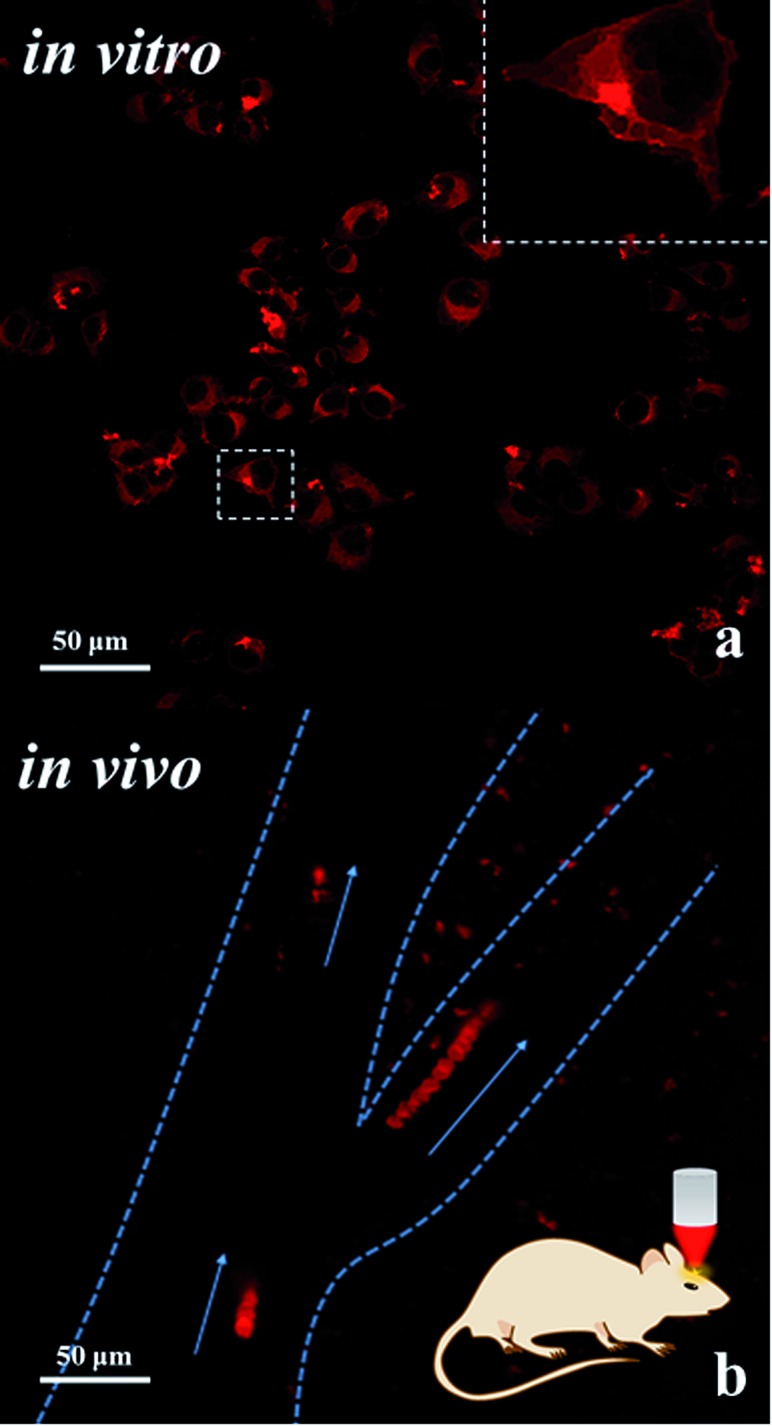
(a) Two-photon laser confocal scanning microscopy (TPLCSM) image of fibroblast cells incubated with ISD-7 upon excitation at 780 nm; the inset shows the high resolution image. (b) TPLCSM image of the blood plasma labelled with the tracer of ISD-7 emulsion in blood vessels; the image shows the flow trajectory by merging the dynamic photo micrographs (the video is included in the ESI, Video-S2†). The blood vessels are indicated by the dashed lines, and the arrows present the flow direction.

The large *δ* of molecule ISD-7 is remarkable considering the simple molecular structure. Importantly, its TPA absorption peaks at 780 nm, which matches well the efficient output of the Ti:sapphire laser and the optical window in biological tissue.^16^ Moreover, ISD-7 exhibits low cytotoxicity (Fig. S4†). To examine its luminescence imaging capability *in vitro*, fibroblast cells were incubated with ISD-7, and then imaged using two-photon laser confocal scanning microscopy (TPLCSM). Intense intracellular luminescence was observed with femtosecond laser pluses at 780 nm (Fig. 6a), under which excitation no background fluorescence from the fibroblast cells would interfere. Benefited from the high resolution of the TPA microscopic image, much detailed intracellular structural information can be obtained. It is found that the luminescence was mainly from the phospholipid bilayer membrane of cytoplasm whereas the nucleus gave no luminescence emission. Though there are very few ISD-7 molecules in the membrane, the membrane structure can be observed in high resolution (inset of Fig. 6a) because of the high *δ* value.

Though NIR excitation is favorable for biological imaging due to the weak absorption by tissue in this wavelength region, the two-photon excited fluorescence (TPEF) intensity diminishes exponentially as the focus deepens into the tissue. Hence fluorescent dyes with large TPA cross section are in great need for *in vivo* imaging. The fluorescent emulsion of ISD-7 was injected into the mice blood vessel by tail vein for *in vivo* tracing of the blood flow. First, the fluorescent emulsion was monitored by the one-photon excited fluorescence microscopy, see video in ESI (Video-S1†). Compared with traditional dyes, the bright red emulsion flows smoothly in the brain vessel in the microscopic video, providing unprecedented contrast to observe both the vessel distribution and the inside fluid situation simultaneously. To track the movements of individual emulsion particles, TPLCSM imaging was performed. The tracking is shown in the ESI (Video-S2†). Twelve dynamic TPLCSM photos are merged in Fig. 6b to indicate the blood flowing behavior. In Fig. 6b, the movements of three separated emulsion particles are marked, which are located in one stem vessel and two branch vessels, respectively. From the tracer of the emulsion particles, it was found that the blood fluid in right branch vessel is faster than the left one and the stem vessel. Such data may provide information for understanding the brain blood flow in different vessels. This result demonstrates that the fluorescent emulsion of ISD-7 can be successfully applied for *in vivo* TPLCSM tracing.

**Fig. 7:**
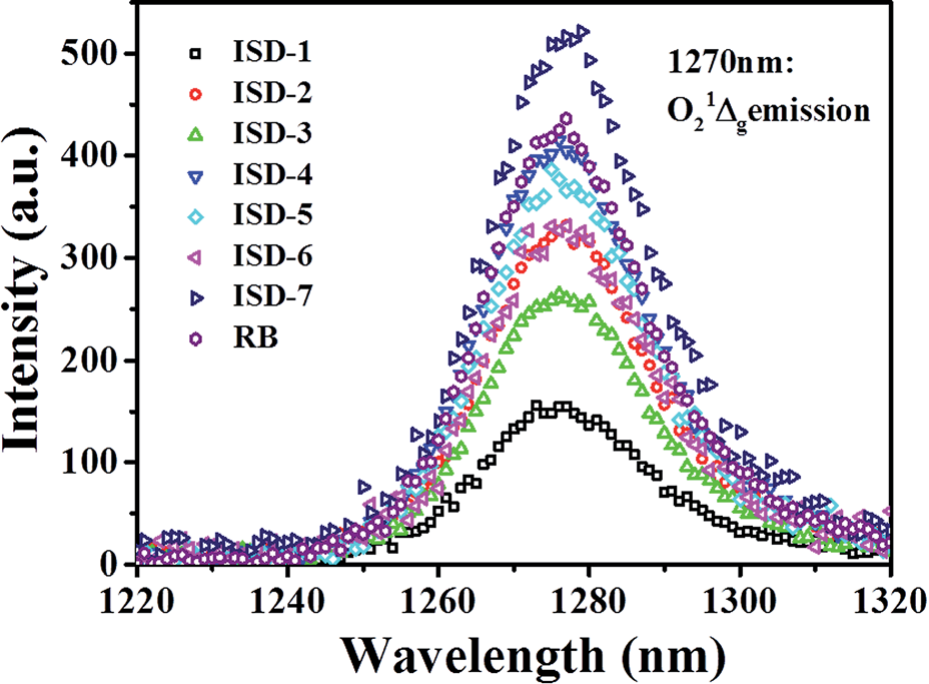
Luminescence of singlet oxygen sensitized with seven ISD dyes and Rose Bengal as reference.

As mentioned above, the designed ISD molecules have a relatively large S_1_–S_0_ energy gap, which is beneficial for photochemical activation. One possible application to take advantage of this property is to activate oxygen and promote generation of singlet oxygen species. To verify this proposal, the singlet oxygen quantum yields (*Φ*_Δ_) of the ISDs were measured by the singlet oxygen luminescent method, using Rose Bengal (RB) as a standard. The singlet oxygen ^1^Δ_g_ emission spectra are shown in Fig. 7 and the determined *Φ*_Δ_ values are collected in Table 1. The *Φ*_Δ_ of ISD-1 is about 0.30, which is normal for this type of dyes. Table 1 shows that all the other ISDs show enhanced *Φ*_Δ_ compared to ISD-1. The *Φ*_Δ_ of Br-containing ISD dyes are larger than their non-Br counterparts, presumably because the heavy Br atom promotes excited-state intersystem crossing.^35^ The *Φ*_Δ_ of ISD-4 is similar to that of other good photosensitizers such as RB. The *Φ*_Δ_ of ISD-7 is above 0.90, which is significantly larger than the other molecules, even larger than RB. Such high *Φ*_Δ_ is very rare among metal-free sensitizers.

Chromophores that have both high *δ* and *Φ*_Δ_ are being considered for two-photon photodynamic therapy (PDT) applications. PDT is a promising noninvasive treatment for cancers and other diseases through singlet oxygen produced by the photosensitizer. Two-photon PDT is advantageous over the traditional one-photon counterpart, because of its deeper penetration capability into living tissues and 3D selectivity. For two-photon PDT applications, the product of *δΦ*_Δ_ describes the efficiency of molecules for two photon singlet oxygen generation. As shown Table 1, the *δΦ*_Δ_ of ISD-7 is as high as 7217, which is the largest for known squaraine dyes. This result indicates that ISD-7 has potential application for two-photon PDT. The relevant biological research is currently under way.

## Experimental

### Calculations

All the calculations were performed with Gaussian 09 software package.^28^ Adiabatic TD-DFT in the Kohn-Sham (KS) form was used for calculating the excited-state structures.^29a,36^ The ground states of all studied molecules were optimized using the density functional theory (DFT) with the B3LYP functional (DFT/B3LYP/6-31G++(d, p)), and the excitation energies were calculated using the time-dependent density functional theory (TD-DFT) at the B3LYP functional (DFT/B3LYP/6-311G++(2d, 2p)). Natural transition orbitals (NTO)^27*a*^ calculations were performed after TD-DFT to describe the physical meanings of the orbitals of the hole and electron on the excitation states. Inhomogeneous line broadening parameter for all absorption spectra calculations has been fixed to *Γ* = 0.17 eV for all chromophores.

### Synthesis

The synthesis and structural characterization of the ISD molecules are provided in the ESI†.

### Absorption and fluorescence spectra

Steady-state absorption spectra were recorded using a T6 UV-Vis spectrometer (Purkinje General, China). Fluorescence measurements were performed on a LS55 fluorescence spectrometer (PerkinElmer, USA). The solutions were bubbled with N_2_ for 10 min before fluorescence measurement. Absolute fluorescence quantum yields (*Φ*) of ISD-1 (0.01 mM in THF) were measured by a FLS920 fluorescence spectrometer using an integrating sphere (Edinburgh, UK). Relative fluorescence quantum yield was measured in THF solution with ISD-1 in THF as reference, using the relative methodology based on the following equation: *Φ*_x_/*Φ*_r_ = (*A*_r_/*A*_x_)(*D*_x_/*D*_r_)(*n*_x_/*n*_r_).^2^ where *A* is the absorbance at the excitation wavelength, *n* the refractive index and *D* the integrated luminescence intensity; “r” and “x” stand for reference and sample.

For the measurements of the fluorescence time profiles, the time-correlated single-photon counting (TCSPC) method using a nanosecond pulsed LED sources (464 nm, FWHM *ca.* 800 ps) with 40 MHz repetition rate was employed. A photomultiplier tube and a counting board (PicoQuanta, PicoHarp 300, Germany) were used for signal detection.

### Singlet oxygen quantum yields (*Φ*_Δ_)

The *Φ*_Δ_ was estimated by the singlet oxygen luminescent method. Singlet-oxygen phosphorescence (near 1270 nm) were recorded using a liquid-nitrogen cooled, solid indium/gallium/arsenic detector (Edinburgh, UK), and *Φ*_Δ_ were deduced from an analogous methodology as similar for fluorescence quantum yields (see above) using Rose Bengal as a reference (*Φ*_Δ_ = 0.76 in methanol).^35,37^

It is noted that one should not be confused by the different values of *Φ* and *Φ*_Δ_ as they corresponding to different processes and were measured under different conditions. The solution concentration for the *Φ*_Δ_ measurement was 0.2 mM, which was much higher than that used for the *Φ* measurement. Besides, all the *Φ*_Δ_ were measured under air-saturation condition. According to the literature,^38^ uncertainties for the quantum yields based on relative luminescence method were 5–10%.

### TPA cross-section

TPA cross-sections were determined *via* a comparative method, by measuring the two-photon excited fluorescence (TPEF) using Rhodamine B as a reference. The fundamental of a mode-locked Ti:sapphire laser (690–850 nm, Tsunami) was focused into a quartz cuvette having an optical geometry, and detected with a liquid-nitrogen cooled charge-coupled device (CCD) (SPEC-10-400 B/LbN, Roper Scientific) attached to a polychromator (Spectropro-550i, Acton).

### Cell culture

Fibroblasts were cultured in Dulbecco's Modified Eagle Medium (Gibco) containing 10% calf bovine serum and 1% penicillin/streptomycin at 37 °C in a humidified atmosphere composed of 95% air and 5% CO_2_. Cells were removed with a solution of 0.05% trypsin in 0.53 mM EDTA, re-suspended in serum-free medium (100 000 cells per mL) for cell seeding, and allowed 2 h to attach to the surface prior to the addition of serum-containing media. For passage, cells were re-suspended in the same 10 mL of medium that they were growing in, and 3 mL was transferred to 7 mL of fresh medium in a new flask. Cells were seeded into a Petri dish, 12-well plate, for different experiments.

### Cell staining

Cells were cultured on 12-well glass slides under normal culture conditions. After a 48 h incubation period, medium was removed. Then cells were incubated with 0.05% ISD-7 in DMSO for 10 min, followed by two washes with PBS. The glass slides were viewed by two-photon confocal fluorescence microscopy.

### Fluorescent emulsion

ISD-7 (1.5 mg) was dissolved in benzyl benzoate (200 μL), then Tween-20 (15 μL) and sterilization water (5 mL) was added and shaken to produce a soft red fluorescent emulsion.

### Animal studies

Mice, wild-type C57BL/6, aged 8–10 weeks and weighing 18–24 g were used throughout the study. All animals were bred in-house and maintained in an aseptic environment supplied with clean water and rodent chow *ad libitum*. Mice were maintained under ketamine anesthesia for the entire imaging session. Animals were fitted into a custom-made two-photon microscope as previously described.^39^ The emulsion of ISD-7 (benzyl benzoate, tween-20 and water) was injected by tail intravenous. At first the fluorescence microscopy was used to observe the emulsion flowing. The video was recorded and included as attachment in ESI (Video-S1†). All experimental procedures and protocols in the study were approved by the Ethics Committee of Lanzhou University, China.

### Two-photon fluoresce microscopy imaging

Cell were visualized with UPlan SApo × 20/0.75 objectives. The cell images were captured sequentially on an Olympus FluoView FV1000 confocal laser scanning microscope (Olympus) with 780 nm excited light. Stacks of 20 optical sections (1024 × 1024 pixel arrays) were collected at 1 μm intervals in the *z* dimension. The image was merged and processed by ImageJ software.

For *in vivo* time-lapse imaging of emulsion flowing, snap (1024 × 1024 pixel arrays) were taken with the emulsion flowing (Video-S2†).

## Conclusions

We have demonstrated a simple and straightforward theoretical approach for screening small organic molecules with large *δ*. We found that the analysis to the morphology of NTOs directly related to the TPA final state can help to qualitatively analysis the TPA performance of indolic squaraine derivatives. Compared with previous theoretical approaches, the NTO analysis method is easy to use and computationally inexpensive. Aided by this method, one small ISD molecule with *δ* above 8000 GM was successfully designed and synthesized. The maximum TPA absorption of this molecule occurs at 780 nm, which matches the commercial laser sources commonly used for TPA microscope. The newly designed TPA dye has excellent performance in both *in vitro* cell labelling and *in vivo* cerebrovascular blood fluid tracing applications. Because of its relatively short TPA absorption wavelength, the ISD-7 dye has a high excitation energy, and exhibits very high singlet oxygen generation efficiency. The rare feature of having both high TPA cross section and high singlet oxygen generation quantum yields makes ISD-7 a potential photosensitizer for two-photon photodynamic therapy (PDT) applications.

We also realize that there are still some limitations of the NTO analysis methodology. For example, the information from the simulation is qualitative and the relationship between the NTOs and the molecule structure needs more definitive description. Our further endeavors will focus on addressing these issues. After all, it is believed that this new theory-assisted molecular design strategy demonstrated in this work will significantly accelerate the discovery of new organic TPA dyes with even further improved performance.

## References

[cit1] Göppert-Mayer M. (1931). Ann. Phys..

[cit2] Spangler C. W. (1999). J. Mater. Chem..

[cit3] Helmchen F., Denk W. (2005). Nat. Methods.

[cit4] Beverina L., Crippa M., Landenna M., Ruffo R., Salice P., Silvestri F., Versari S., Villa A., Ciaffoni L., Collini E., Ferrante C., Bradamante S., Mari C. M., Bozio R., Pagani G. A. (2008). J. Am. Chem. Soc..

[cit5] LaFratta C. N., Fourkas J. T., Baldacchini T., Farrer R. A. (2007). Angew. Chem., Int. Ed..

[cit6] Parthenopoulos D. A., Rentzepis P. M. (1989). Science.

[cit7] Pawlicki M., Collins H. A., Denning R. G., Anderson H. L. (2009). Angew. Chem., Int. Ed..

[cit8] Chung S. J., Zheng S., Odani T., Beverina L., Fu J., Padilha L. A., Biesso A., Hales J. M., Zhan X., Schmidt K., Ye A., Zojer E., Barlow S., Hagan D. J., Van Stryland E. W., Yi Y., Shuai Z., Pagani G. A., Bredas J. L., Perry J. W., Marder S. R. (2006). J. Am. Chem. Soc..

[cit9] Bort G., Gallavardin T., Ogden D., Dalko P. I. (2013). Angew. Chem., Int. Ed..

[cit10] Collins H. A., Khurana M., Moriyama E. H., Mariampillai A., Dahlstedt E., Balaz M., Kuimova M. K., Drobizhev M., Yang V. X. D., Phillips D., Rebane A., Wilson B. C., Anderson H. L. (2008). Nat. Photonics.

[cit11] Yao S., Belfield K. D. (2012). Eur. J. Org. Chem..

[cit12] Beverina L., Fu J., Leclercq A., Zojer E., Pacher P., Barlow S., Van Stryland E. W., Hagan D. J., Bredas J. L., Marder S. R. (2005). J. Am. Chem. Soc..

[cit13] Williams-Harry M., Bhaskar A., Rarnakrishna G., Goodson III T., Imamura M., Mawatari A., Nakao K., Enozawa H., Nishinaga T., Iyoda M. (2008). J. Am. Chem. Soc..

[cit14] Drobizhev M., Stepanenko Y., Rebane A., Wilson C. J., Screen T. E. O., Anderson H. L. (2006). J. Am. Chem. Soc..

[cit15] Lakowicz J. and Gryczynski I., in Topics of Fluorescence Spectroscopy: Nonlinear and Two-Photon-Induced Fluorescence, New York, 1997.

[cit16] Beverina L., Salice P. (2010). Eur. J. Org. Chem..

[cit17] Albota M., Beljonne D., Bredas J. L., Ehrlich J. E., Fu J. Y., Heikal A. A., Hess S. E., Kogej T., Levin M. D., Marder S. R., McCord-Maughon D., Perry J. W., Rockel H., Rumi M., Subramaniam C., Webb W. W., Wu X. L., Xu C. (1998). Science.

[cit18] Orr B. J., Ward J. F. (1971). Mol. Phys..

[cit19] Cohen H. D., Roothaan C. C. J. (1965). J. Chem. Phys..

[cit20] Masunov A., Tretiak S. (2004). J. Phys. Chem. B.

[cit21] Arnbjerg J., Jimenez-Banzo A., Paterson M. J., Nonell S., Borrell J. I., Christiansen O., Ogilby P. R. (2007). J. Am. Chem. Soc..

[cit22] Ajayaghosh A. (2005). Acc. Chem. Res..

[cit23] Ohira S., Rudra I., Schmidt K., Barlow S., Chung S. J., Zhang Q., Matichak J., Marder S. R., Bredas J. L. (2008). Chem.–Eur. J..

[cit24] Odom S. a., Webster S., Padilha L. A., Peceli D., Hu H., Nootz G., Chung S.-J., Ohira S., Matichak J. D., Przhonska O. V., Kachkovski A. D., Barlow S., Brédas J.-L., Anderson H. L., Hagan D. J., Van Stryland E. W., Marder S. R. (2009). J. Am. Chem. Soc..

[cit25] Beverina L., Crippa M., Salice P., Ruffo R., Ferrante C., Fortunati I., Signorini R., Mari C. M., Bozio R., Facchetti A., Pagani G. A. (2008). Chem. Mater..

[cit26] Heflin J. R., Wong K. Y., Zamani-Khamiri O., Garito A. F. (1988). Phys. Rev. B.

[cit27] Martin R. L. (2003). J. Chem. Phys..

[cit28] Gaussian R. A., Frisch M. J., Trucks G. W., Schlegel H. B., Scuseria G. E., Robb M. A., Cheeseman J. R., Scalmani G., Barone V., Mennucci B., Petersson G. A., Nakatsuji H., Caricato M., Li X., Hratchian H. P., Izmaylov A. F., Bloino J., Zheng G., Sonnenberg J. L., Hada M., Ehara M., Toyota K., Fukuda R., Hasegawa J., Ishida M., Nakajima T., Honda Y., Kitao O., Nakai H., Vreven T., Montgomery Jr J. A., Peralta J. E., Ogliaro F., Bearpark M., Heyd J. J., Brothers E., Kudin K. N., Staroverov V. N., Kobayashi R., Normand J., Raghavachari K., Rendell A., Burant J. C., Iyengar S. S., Tomasi J., Cossi M., Rega N., Millam J. M., Klene M., Knox J. E., Cross J. B., Bakken V., Adamo C., Jaramillo J., Gomperts R., Stratmann R. E., Yazyev O., Austin A. J., Cammi R., Pomelli C., Ochterski J. W., Martin R. L., Morokuma K., Zakrzewski V. G., Voth G. A., Salvador P., Dannenberg J. J., Dapprich S., Daniels A. D., Farkas Ö., Foresman J. B., Ortiz J. V., Cioslowski J. and Fox D. J., Gaussian, Inc., Wallingford, CT, 2009.

[cit29] Furche F., Ahlrichs R. (2002). J. Chem. Phys..

[cit30] Xu Z.-G., Wu G.-P., Wang L.-J., Sun C.-L., Shi Z.-F., Zhang H.-L., Wang Q. (2011). Chem. Phys. Lett..

[cit31] Small G. J. (1971). J. Chem. Phys..

[cit32] Chandra A. K., Turro N. J., Lyons A. L., Stone P. (1978). J. Am. Chem. Soc..

[cit33] (a) Malkin J., Photophysical and Photochemical Properties of Aromatic Compounds, CRC Press, Boca Raton, FL, 1992.

[cit34] Mayerhöffer U., Fimmel B., Würthner F. (2012). Angew. Chem., Int. Ed..

[cit35] Gallavardin T., Armagnat C., Maury O., Baldeck P. L., Lindgren M., Monnereau C., Andraud C. (2012). Chem. Commun..

[cit36] Jamorski C., Casida M. E., Salahub D. R. (1996). J. Chem. Phys..

[cit37] McCormick T. M., Calitree B. D., Orchard A., Kraut N. D., Bright F. V., Detty M. R., Eisenberg R. (2010). J. Am. Chem. Soc..

[cit38] Wuerth C., Grabolle M., Pauli J., Spieles M., Resch-Genger U. (2013). Nat. Protoc..

[cit39] Li T., Pang S., Yu Y., Wu X., Guo J., Zhang S. (2013). Brain.

