# Supplementary material for: Rational design of small indolic squaraine dyes with large two-photon absorption cross section
Source: Chem Sci. 2014 Oct 7;6(1):761–9. doi: 10.1039/c4sc02165g (PMC5590541; doi:10.1039/c4sc02165g)
Supplement: Supplementary file 1 [file SC-006-C4SC02165G-s001.pdf]

Electronic Supplementary Material (ESI) for Chemical Science.
This journal is © The Royal Society of Chemistry 2014

Video-S1

Movie of fluorescent emulsion containing ISD-7 moving in the mice brain vessel recorded by one-photon excited fluorescence microscopy.

Video-S2

Moving of emulsion containing ISD-7 in the mice brain vessel monitored by two-photon laser confocal scanning microscopy.
