# Supplementary material for: Rational design of small indolic squaraine dyes with large two-photon absorption cross section
Source: Chem Sci. 2014 Oct 7;6(1):761–9. doi: 10.1039/c4sc02165g (PMC5590541; doi:10.1039/c4sc02165g)
Supplement: Supplementary file 2 [file SC-006-C4SC02165G-s002.pdf]

Electronic Supplementary Material (ESI) for Chemical Science.
This journal is © The Royal Society of Chemistry 2014

Rational Design of Small Indolic Squaraine Dyes with Large Two-photon Absorption Cross Section

Chun-Lin Sun[a], Qing Liao[b], Ting Li[c], Jun Li[a], Jian-Qiao Jiang[a], Zhen-Zhen Xu[b], Xue-Dong Wang[b], Rong Shen[d], De-Cheng Bai[d], Qiang Wang[a], Sheng-Xiang Zhang[c], Hong-Bing Fu[b,e], Hao-Li Zhang[*,a]

[a] State Key Laboratory of Applied Organic Chemistry (SKLAOC), College of Chemistry and Chemical Engineering, Lanzhou University, Lanzhou 73000, P. R. China,

E-mail: haoli.zhang@lzu.edu.cn

[b]Beijing National Laboratory for Molecular Sciences (BNLMS) Institute of chemistry, Chinese Academy of Sciences, Beijing 100190, P. R. China

[c]School of Life Sciences, Lanzhou University, Lanzhou 73000, P. R. China

[d]School of Basic Medical Sciences, Lanzhou University, Lanzhou 730000, P. R. China

[e]Department of Chemistry, Capital Normal University, Beijing 100048, P. R. China

Table of contents

1. **Molecule synthesis and characterization**

2. **The natural transition orbitals (NTOs) of the S_1 and S_2 states (Fig. S1)**

3. **Digital photo of ISDs in solution (Fig. S2)**

4. **The time-resolved fluorescence spectra (Fig. S3)**

5. **The cytotoxicity of the ISD-7 molecule (Fig. S4)**

6. **Other ISD molecues studied theoretically (Fig. S5)**

7. **References**

1. Molecule synthesis and characterization

All the reagents and materials were purchased from J&K Chemical Co. (China), and are of analytical grade. The solvents were purified by the standard procedures. NMR(^{1}H, ^{13}C) spectra were recorded on a Mercury Plus 300BB 300MHz, Bruker ADVANCE III 400MHz or INOVA 600MHz spectrometer at room temperature. The ^{1}H and ^{13}C chemical shifts (δ) are reported in parts per million and the TMS was used as an internal standard, Mass spectra were obtained on Esquire6000 Bruker Daltonics iron trap mass spectrometer. Elemental analysis was carried out using vario EL cube elemental analyzer.

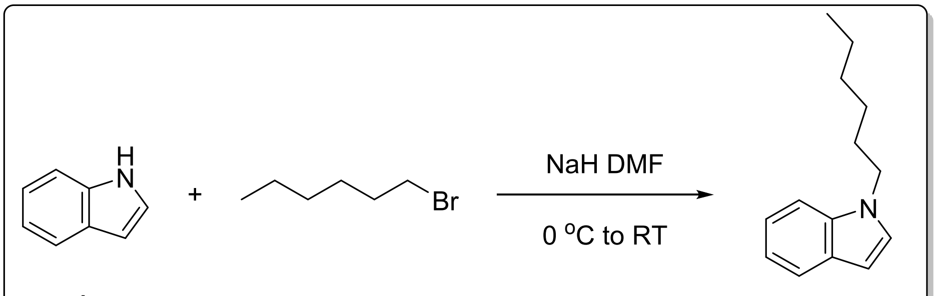

1a 1b

1-hexyl-1H-indole (1b): To a stirred solution of indole **(1a)** (292.9 mg, 2.50 mmol) in dry DMF (6.0 mL), NaH (120.0mg, 3.00 mmol) was added portionwise under nitrogenatmosphere at 0 °C. The reaction mixture was then warmed to room temperature and stirred for 30 min. After cooling to 0 °C, 1-bromohexane (0.22 mg, 3.00 mmol) was added dropwise to the reaction mixture. The reaction mixture was warmed to room temperature and stirred overnight. Water was added and the aqueous layer was extracted with ether. The combined organic layers were washed with brine, dried over anhydrous Na_2SO_4 and concentrated under reduced pressure. The residue was purified by column chromatography on silica gel (*n*-hexane/ethyl acetate = 15/1) to give compound **1b** as a yellow oil (478.1 mg, 95% yield). ^{1}H NMR (300 MHz, $CDCl_3$): *d* = 0.89 (t, *J* = 6.9 Hz, 3H), 1.27-1.37 (m, 6H), 1.85 (quint, *J* =7.2 Hz, 2H), 4.13 (t, *J* = 7.1 Hz, 2H), 6.51 (d, *J* = 3.2 Hz, 1H), 7.09-7.15 (m, 2H), 7.19-7.26 (m, 1H), 7.37 (d, *J* = 8.3 Hz, 1H), 7.66 ppm (d, *J* = 7.8 Hz, 1H). MS (ESI, *m/z*) 202.2 $[M+H]^+$. These assignments matched with those previously published.[1]

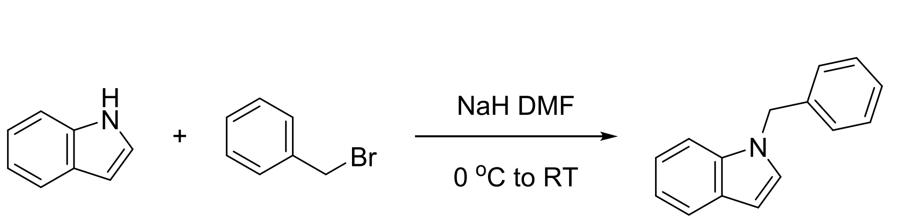

1a 2b

1-benzyl-1H-indole (2b): To a stirred solution of indole **1a** (292.9 mg, 2.50 mmol) in dry DMF (6.0 mL), NaH (120.0mg, 3.00 mmol) was added portionwise under nitrogen atmosphere at 0 °C. The reaction mixture was then warmed to room temperature and stirred for 30 min. After cooling to

0 °C, BnBr (513.0 mg, 3.00 mmol) was added dropwise to the reaction mixture. The reaction mixture was warmed to room temperature and stirred overnight. Water was added and the aqueous layer was extracted with ether. The combined organic layers were washed with brine, dried over anhydrous Na_2SO_4 and concentrated under reduced pressure. The residue was purified by column chromatography on silica gel (*n*-hexane/ethyl acetate = 30/1) to give compound **3b** as a clear oil (461.2 g, 89% yield). ^{1}H NMR ($CDCl_3$, 300 MHz) δ 5.32 (s, 2H), 6.55 (dd, *J* = 3.3 Hz, *J* = 0.9 Hz, 1H), 7.09-7.29 (m, 9H), 7.65 (m, 1H); MS (ESI, *m/z*) 208.1 $[M+H]^+$. These assignments matched with those previously published.[2]

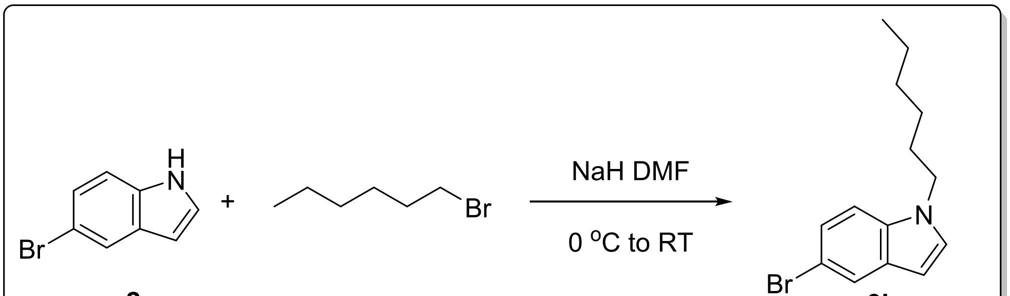

2a 3b

5-bromo-1-hexyl-1H-indole (3b): To a stirred solution of 5-bromo-1H-indole **2a** (490.0 mg, 2.50 mmol) in dry DMF (6.0 mL), NaH (120.0mg, 3.00 mmol) was added portionwise under nitrogenatmosphere at 0 °C. The reaction mixture was then warmed to room temperature and stirredfor 30 min. After cooling to 0 °C, 1-bromohexane (0.22 mg, 3.00 mmol) was added dropwise to the reaction mixture. The reaction mixture was warmed to room temperature and stirred overnight. Water was added and the aqueous layer was extracted with ether. The combined organic layers were washed with brine, dried over anhydrous Na_2SO_4 and concentrated under reduced pressure. The residue was purified by column chromatography on silica gel (*n*-hexane/ethyl acetate = 15/1) to give compound **3c** as a white solid (644.5 mg, 92% yield). ^{1}H NMR ($CDCl_3$) *δ* (ppm): 7.67 (s, 1H), 7.20 (d, *J* = 8.7 Hz, 1H), 7.20 (d, *J* = 8.7 Hz,1H), 7.02 (d, *J* = 2.1 Hz, 1H), 6.35(s, 1H), 4.01 (t, *J* = 7.5 Hz, 2H), 1.74 (d, *J* = 7.2 Hz, 2H), 1.23-1.34 (m, 6H), 0.80 (t, *J* = 6.8 Hz, 3H), MS (ESI, *m/z*) 280.1 $[M+H]^+$. These assignments matched with those previously published.[3]

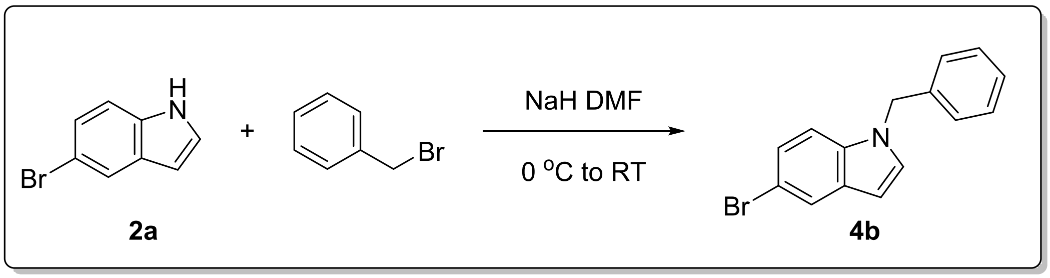

2a 4b

1-benzyl-5-bromo-1H-indole (4b): To a stirred solution of 5-bromo-1H-indole **2a** (490.0 mg, 2.50 mmol) in dry DMF (6.0 mL), NaH 120.0mg, 3.00 mmol) was added portionwise under

nitrogen atmosphere at 0 °C. The reaction mixture was then warmed to room temperature and stirred for 30 min. After cooling to 0 °C, BnBr (513.0 mg, 3.00 mmol) was added dropwise to the reaction mixture. The reaction mixture was warmed to room temperature and stirred overnight. Water was added and the aqueous layer was extracted with ether. The combined organic layers were washed with brine, dried over anhydrous Na_2SO_4 and concentrated under reduced pressure. The residue was purified by column chromatography on silica gel (*n*-hexane/ethyl acetate = 30/1) to give compound **4b** as a white solid (3.4152 g, 92% yield). ^{1}H NMR (CDCl3, 300 MHz) δ 5.32 (s, 2H), 6.55 (dd, J^1 = 3.3 Hz, J^2 = 0.9 Hz, 1H), 7.09-7.29 (m, 9H), 7.65 (m, 1H); MS (ESI, *m/z*) 286.0 $[M+H]^+$. These assignments matched with those previously published.[4]

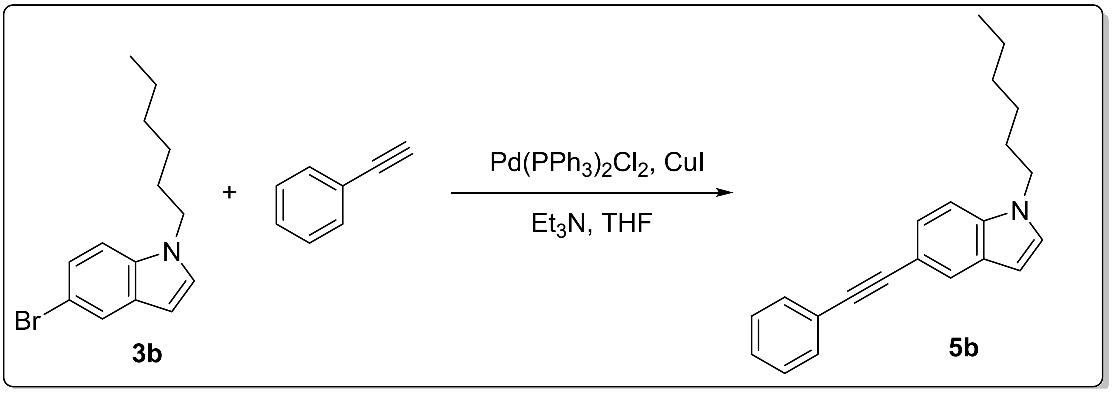

1-hexyl-5-(phenylethynyl)-1H-indole (5b): A dried and nitrogenpurged reaction tube was loaded with 5-bromo-1-hexyl-1H-indole **3b** (420.3 mg, 1.5 mmol), $Pd(PPh_3)_2Cl_2$ (52.6 mg, 0.075 mmol), copper(I) iodide (28.6 mg, 0.15 mmol) and was then evacuated and refilled with Argon. THF (20 mL), triethylamine (3 mL), and phenylacetylene (183.8 mg,1.8 mmol) were then added by syringe, and the brown suspension was stirred for 18 h at 60 °C. After complete consumption of the starting material (TLC monitoring) the brown solution was diluted with ethyl acetate (50 mL) and filtered, and the solvents were evaporated to dryness. Purification by flash chromatography on silica (pentane/ethyl acetate, 40:1) gave product **5b** as a yellow solid (397.8 mg, 88%); ^{1}H NMR (400M, $CDCl_3$) 1.06 (t, J = 7.0 Hz, 2H), 1.45-1.48 (m, 6H), 1.98-2.02 (m, 2H), 4.27 (t, J = 7.2 Hz, 2H), 6.61 (d, J = 3 Hz, 1H), 7.28 (t, J = 3.0 Hz 1H), 7.38-7.58 (m, 5H), 7.73 (dd, J^1 = 7.2 Hz, J^2 = 1.5 Hz, 2H), 7.93 (d, J = 1.2 Hz, 1H);

^{13}C NMR (100M, $CDCl_3$) 14.2, 22.7, 26.8, 30.4, 31.6, 46.8, 82.0, 83.8 ,100.6, 111.0, 112.7, 122.7, 123.5, 124.3, 128.7, 129.1, 129.4, 130.4, 132.7, 136.7;

MS (ESI+) 302.2 $[M+1]^+$。

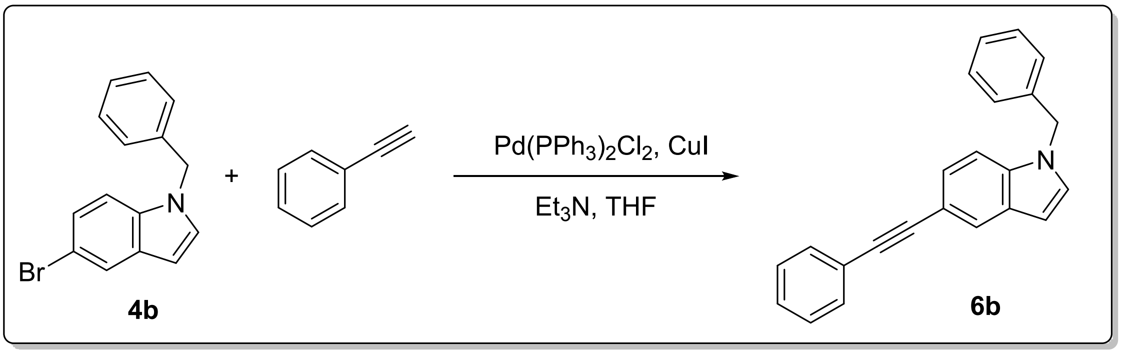

1-benzyl-5-(phenylethynyl)-1H-indole (6b): A dried and nitrogenpurged reaction tube was loaded with 1-benzyl-5-bromo-1H-indole **4b** (429.3 mg, 1.5 mmol), $Pd(PPh_3)_2Cl_2$ (52.6 mg, 0.075 mmol), copper(I) iodide (28.6 mg, 0.15 mmol) and was then evacuated and refilled with Argon. THF (20 mL), triethylamine (3 mL), and phenylacetylene (183.8 mg,1.8 mmol) were then added by syringe, and the brown suspension was stirred for 18 h at 60 ^{o}C. After complete consumption of the starting material (TLC monitoring) the brown solution was diluted with ethyl acetate (50 mL) and filtered, and the solvents were evaporated to dryness. Purification by flash chromatography on silica (pentane/ethyl acetate, 40:1) gave product **6b** as a yellow solid (391.9 mg, 85%);

^{1}H NMR (400M, $CDCl_3$) 1.06 (t, J = 7.0 Hz, 2H), 1.45-1.48 (m, 6H), 1.98-2.02 (m, 2H), 4.27 (t, J = 7.2 Hz, 2H), 6.61 (d, J = 3 Hz, 1H), 7.28 (t, J = 3.0 Hz 1H), 7.38-7.58 (m, 5H), 7.73 (dd, J^1 = 7.2 Hz, J^2 = 1.5 Hz, 2H), 7.93 (d, J = 1.2 Hz, 1H);

^{13}C NMR (100M, $CDCl_3$) 50.2, 74.2, 81.8, 101.4, 111.3, 113.0, 123,5, 124,6, 126.8, 127.8, 128.5, 128.9, 129.6, 130.5, 131.5, 131.7, 132.6, 135.0, 137.1;

MS (ESI+) 308.1 $[M+1]^+$.

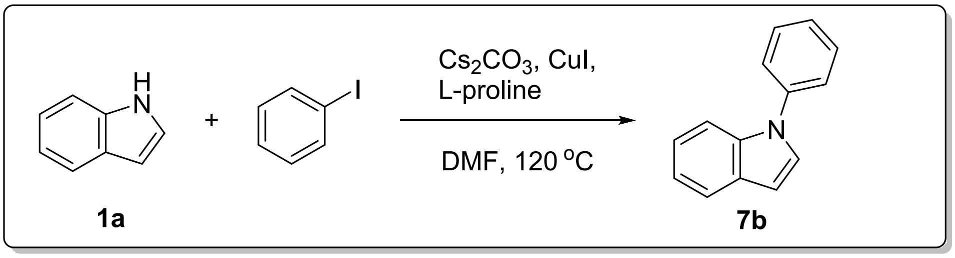

1-phenyl-1H-indole (7b): To a suspension of indole **1a** (292.9 mg, 2.50 mmol), Cs_2CO_3 (1.222 g, 3.75 mmol), CuI (47.6 mg, 0.25 mmol) and L-proline (82.6 mg, 0.50 mmol) in dry DMF (12.0 mL), PhI (637.5 mg, 3.13 mmol) was added under a nitrogen atmosphere. The reaction mixture was heated to 120 ^{o}C overnight. The reaction mixture was allowed to cool to room temperature, diluted with Et_2O, washed with H_2O and brine, dried over Na_2SO_4 and concentrated under reduced pressure. The residue was purified by column chromatography on silica gel (*n*-hexane/ethyl acetate = 30/1) to give compound 3e as a clear oil (391.2 mg, 81% yield). ^{1}H NMR (CDCl3, 300 MHz) δ 6.89 (d, J = 2.7 Hz, 1H), 7.14-7.25 (m, 2H), 7.33-7.39 (m, 2H), 7.51-7.56 (m, 4H), 7.57 (d, J = 8.1

Hz, 1H), 7.69 (d, J = 8.4 Hz, 1H); MS (ESI, *m/z*) 194.1 $[M+H]^+$. These assignments matched with those previously published.[5]

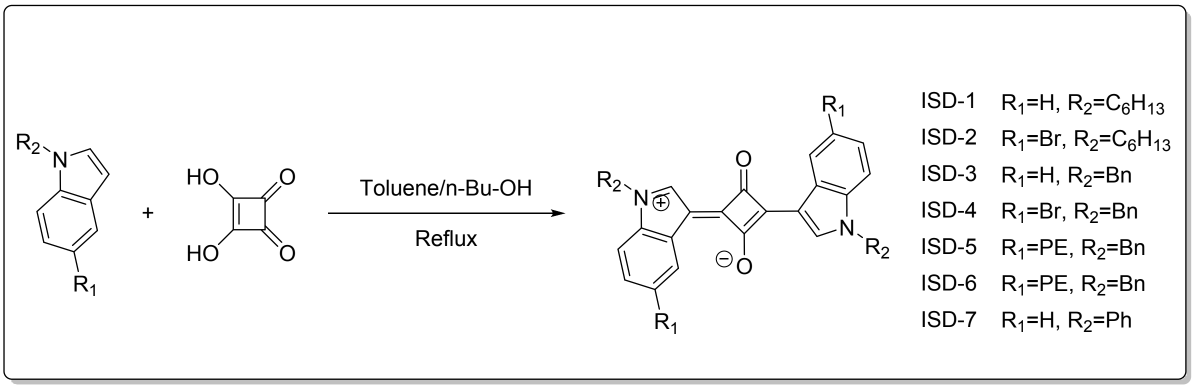

A general procedure for the systheis of indolic squaraine dyes[6]: A suspension of indole derivative (**1b** to **7b**) (1,600 g, 7.33 mmol) and squaric acid (0.468 g, 3.66 mmol) in a 1:1 by volume mixture of BuOH and toluene (100 ml) was refluxed under a Dean-Stark trap for 2 h. Reaction mixture readily turns deep violet. All volatiles were removed by vacuum distillation and and the residue was purified by column chromatography on silica gel (DCM/Methanol = 20/1) to give compound ISD-1 to ISD-7.

ISD-1

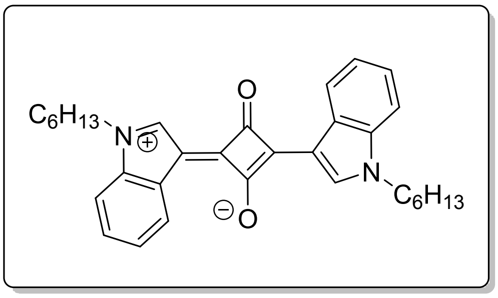

^{1}H NMR (600M, DMSO-d_6) 0.85 (t, J = 6.8 Hz, 6H), 1.25-1.39 (m, 12H), 1.96 (m, 4H), 4.23 (t, J = 7.2 Hz, 4H), 7.32-7.45 (m, 6H), 7.72 (dd, J^1 = 6.5 Hz, J^2 = 2.2 Hz, 2H), 8.85 (s,2H);

^{13}C NMR (150M, DMSO-d_6); 13.9, 22.4, 26.5, 29.5, 31.3, 48.1, 110.6, 111.8 124.1, 124.8,126.7, 127.8, 137.4,137.7, 179.3, 187.4;

MS (ESI+) 481.3 $[M+1]^+$;

Elemental analysis (%) calculated for $C_{32}H_{36}N_2O_2$: C, 79.96; H, 7.55; N, 5.83; found C, 79.81; H, 7.57; N, 5.81;

ISD-2

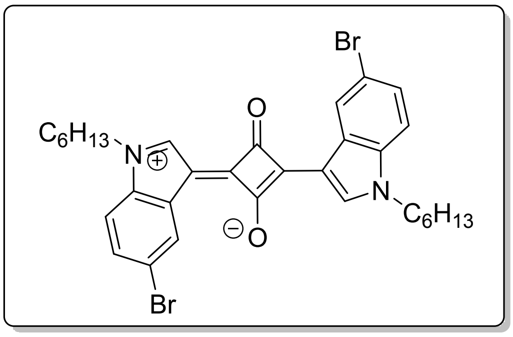

^{1}H NMR (600M, DMSO-d_6); 0.89 (t, J = 6.8 Hz, 6H), 1.26-1.39 (m, 12H), 1.95 (m, 4H), 4.22 (t, J = 6.8 Hz, 4H), 7.24-7.30 (br, 2H), 7.46 (d, J = 6.8 Hz, 2H), 8.65 (s,2H), 8.98 (s, 2H);

^{13}C NMR (150M, DMSO-d_6) 13.9, 22.0, 25.7, 29.2, 30.7, 47.5, 109.9, 113.2, 116.6, 124.9, 126.5, 127.5, 136.7, 139.2, 178.7, 185.8;

MS (ESI+) 637.1 $[M+1]^+$;

Elemental analysis (%) calculated for $C_{32}H_{34}Br_2N_2O_2$: C, 60.20; H, 5.37; N, 4.39; found C, 60.15; H, 5.38; N, 4.37;

ISD-3

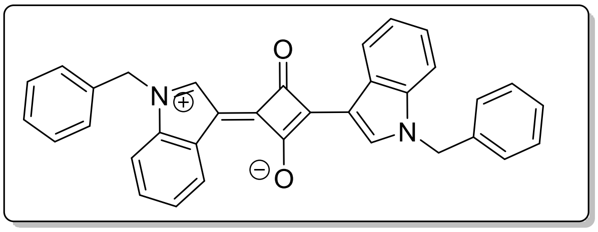

^{1}H NMR (600M, DMSO-d_6) 5.72 (s, 4H), 7.31-7.40 (m, 14H), 7.67 (d, J = 7.2 Hz, 2H), 8.69 (br, 2H), 8.98 (s, 2H);

^{13}C NMR (150M, DMSO-d_6); 50.5, 110.9, 112.4, 123.8, 124.1, 124.9, 126.2, 127.6, 128.1,128.9, 135.9, 137.7, 138.4, 179.9, 187.1;

MS (ESI+) 493.2 $[M+1]^+$;

Elemental analysis (%) calculated for $C_{34}H_{24}N_2O_2$: C, 82.91; H, 4.91; N, 5.69; found C, 82.80; H, 4.92; N, 5.67;

ISD-4

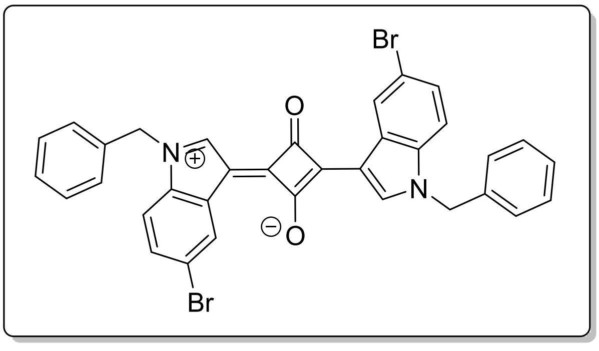

^{1}H NMR (600M, DMSO-d_6) 5.72 (s, 4H), 7.33-7.39 (m, 8H), 7.54 (d, J = 7.2 Hz, 2H), 7.66 (d, J = 8.4 Hz, 2H), 8.69 (s, 2H), 9.00 (s, 2H);

^{13}C NMR (150M, DMSO-d_6); 50.6, 110.2, 114.6, 116.6, 126.4, 127.5, 127.9, 128.2, 128.9, 135.6, 136.6, 139.3, 178.3, 187.1;

MS (ESI+) 649.0 $[M+1]^+$;

Elemental analysis (%) calculated for $C_{34}H_{22}Br_2N_2O_2$: C, 62.79; H, 3.41; N, 4.31; found C, 62.70; H, 3.41; N, 4.28;

ISD-5

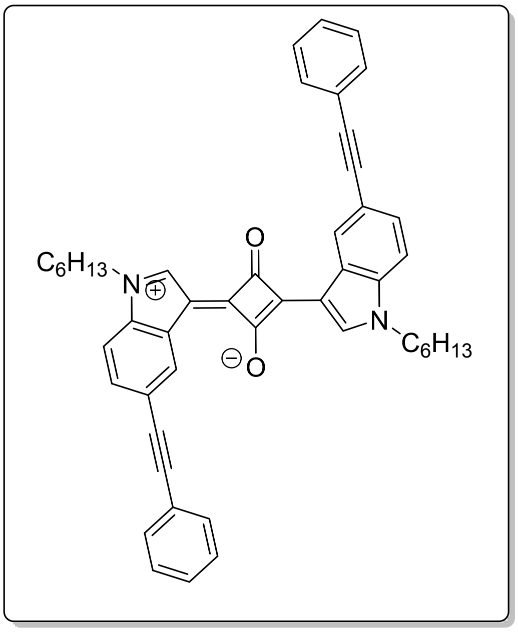

^{1}H NMR (600M, DMSO-d_6) 0.84 (t, J = 7.2 Hz, 6H), 1.22-1.29 (m, 12H), 1.83 (t, J = 7.2 Hz, 4H), 4.44 (t, J = 7.2 Hz, 4H), 7.44-7.51 (m, 6H), 7.56-7.62 (m, 8H), 8.81 (s, 2H), 8.90 (s, 2H);

^{13}C NMR (150M, DMSO-d_6) 13.8, 21.9, 25.6, 29.1, 30.6, 47.4, 73.4, 81.8, 109.8, 114.3, 116.4, 120.4, 126.4, 127.3, 127.7, 128.9, 130.0, 132.4, 136.6, 139.1, 178.4, 188.6;

MS (ESI+) 681.3 $[M+1]^+$;

Elemental analysis (%) calculated for $C_{48}H_{44}N_2O_2$: C, 84.67; H, 6.51; N, 4.11; found C, 84.59; H, 6.54; N, 4.13;

ISD-6

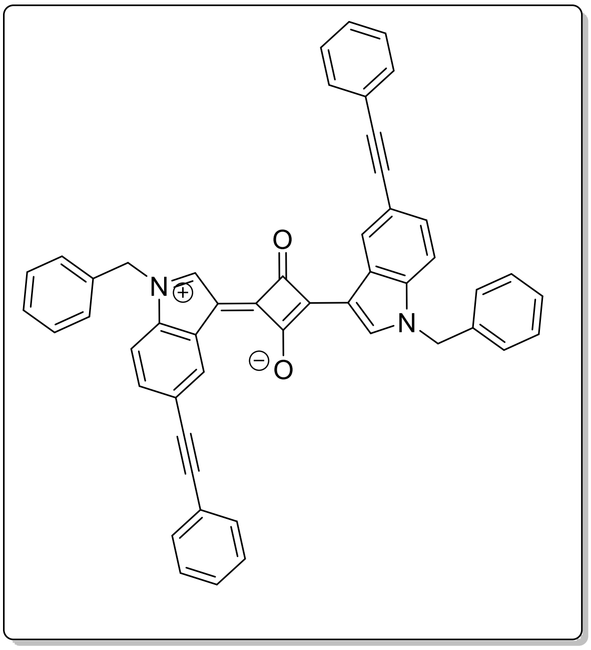

^{1}H NMR (600M, DMSO-d_6) 5.75 (s 4H), 7.25-7.62 (m, 24H), 8.21 (s, 2H), 9.03 (s, 2H);

^{13}C NMR (150M, DMSO-d_6); 49.3, 72.9, 81.2, 110.8, 112.4, 116.3, 124.1, 123.6, 124.8, 126.2, 127.2, 127.6, 128.1, 128.7, 128.9, 131.2, 135.9, 137.7, 138.4, 179.3, 189.1;

MS (ESI+) 693.2 $[M+1]^+$;

Elemental analysis (%) calculated for $C_{50}H_{32}N_2O_2$: C, 86.68; H, 4.66; N, 4.04; found C, 86.55; H, 4.67; N, 4.02;

ISD-7

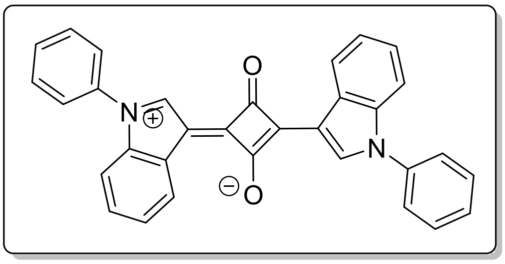

^{1}H NMR (600M, DMSO-d_6) 7.47-7.53 (m, 4H), 7.60 (d, J = 4.2 Hz, 2H), 7.63 (d, J = 7.6 Hz, 2H), 7.71 (dd, J_1 = 8.4 Hz, J_2=4.2 Hz, 4H), 7.82 (d, J = 8.4 Hz, 4H), 8.81 (d, J = 6.0 Hz, 2H), 8.86 (s, 2H);

^{13}C NMR (150M, DMSO-d_6); 103.7, 112.3, 112.4, 124.5, 124.8, 124.8, 125.7, 126.2, 129.1, 130.2, 136.8, 137.7, 178.6, 188.7;

MS (ESI+) 465.2 $[M+1]^+$;

Elemental analysis (%) calculated for $C_{32}H_{20}N_2O_2$: C, 82.74; H, 4.34; N, 6.03; found C, 82.70; H, 4.37; N, 6.05.

2. The natural transition orbitals (NTOs) of the S_1 and S_2 states

<table>
<tr><th>Molecules</th><th></th><th>HOLE</th><th>Particle</th></tr>
<tr><td rowspan="2">ISD-1</td><td>|1>
W=1.01724
f=1.0761
2.4902eV</td><td>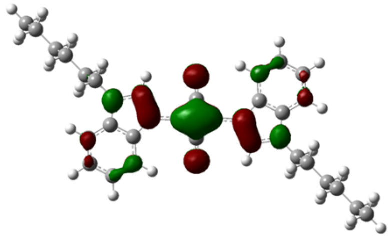</td><td>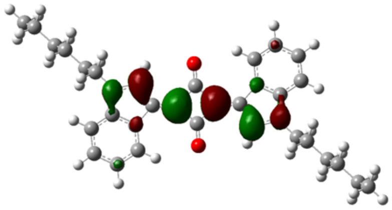</td></tr>
<tr><td>|2>
w=0.99776
f=0.0001
3.3440eV</td><td>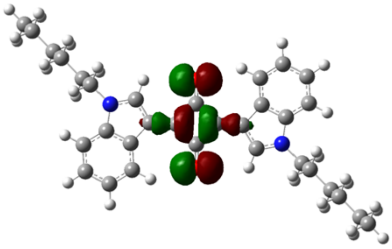</td><td>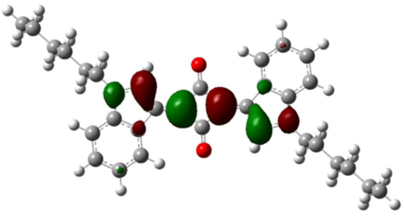</td></tr>
<tr><td rowspan="2">ISD-2</td><td>|1>
w=1.01424
f=1.0590
2.4587eV</td><td>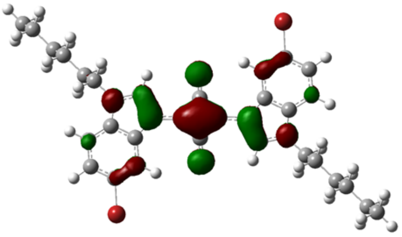</td><td>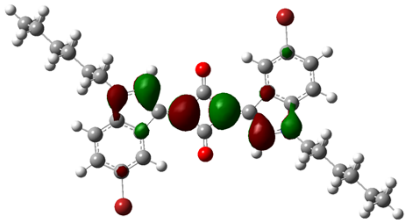</td></tr>
<tr><td>|2>
w=0.98368
f=0.0000
3.1190eV</td><td>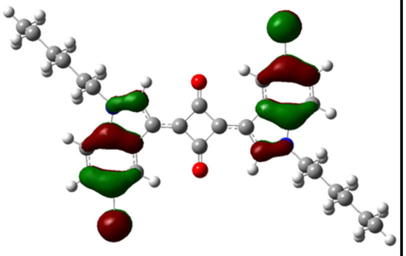</td><td>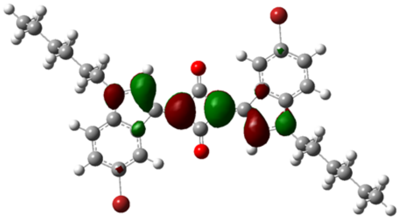</td></tr>
<tr><td rowspan="2">ISD-3</td><td>|1>
w=1.01588
f=1.1270
2.4721eV</td><td>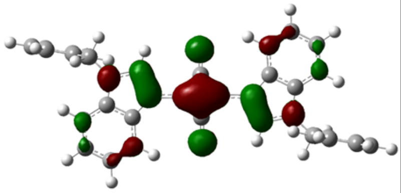</td><td>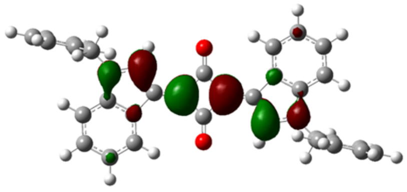</td></tr>
<tr><td>|2>
w=0.95262
f=0.0000
3.3261eV</td><td>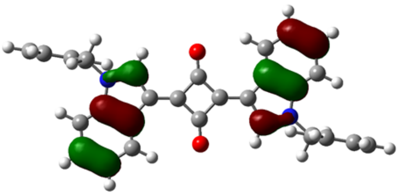</td><td>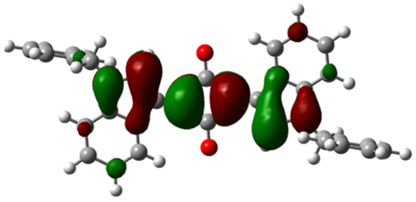</td></tr>
</table>

**ISD-4**	|1><br>w=1.01311<br>f=1.1119<br>2.4444eV	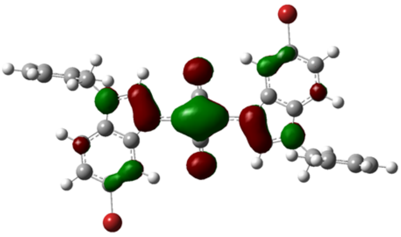	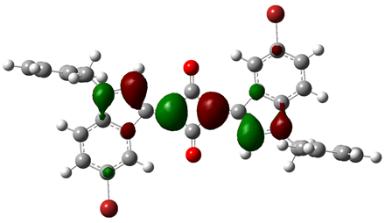
	|2><br>w= 0.98632<br>f=0.0000<br>3.0871eV	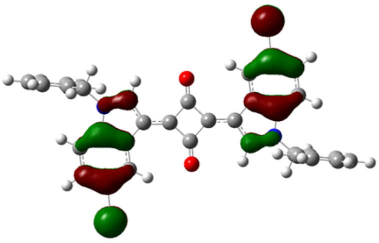	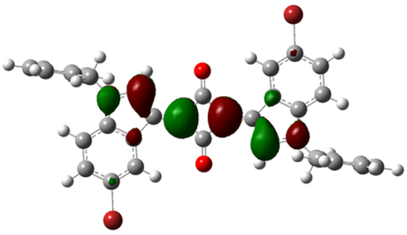
**ISD-5**	|1><br>w=1.00269<br>f=1.0217<br>2.2771eV	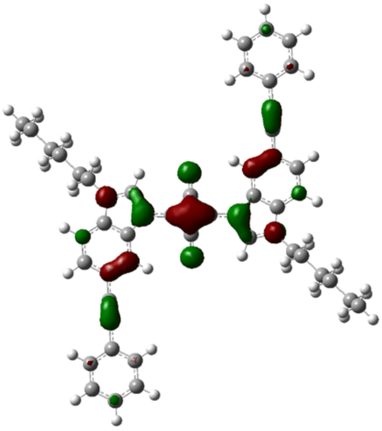	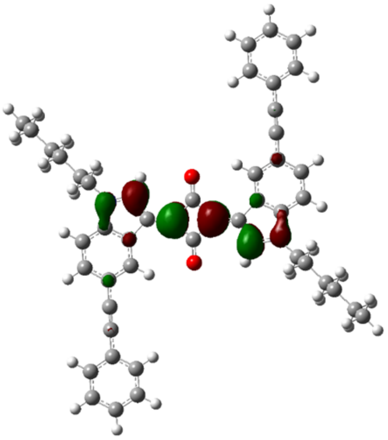
	|2><br>w=0.99543<br>f=0.0000<br>2.5996eV	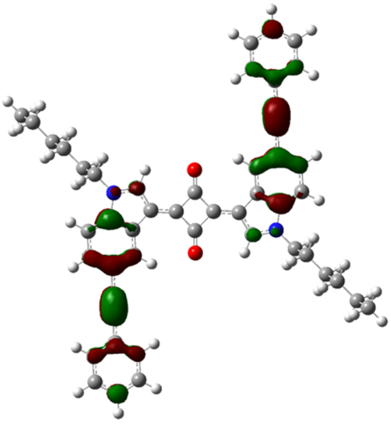	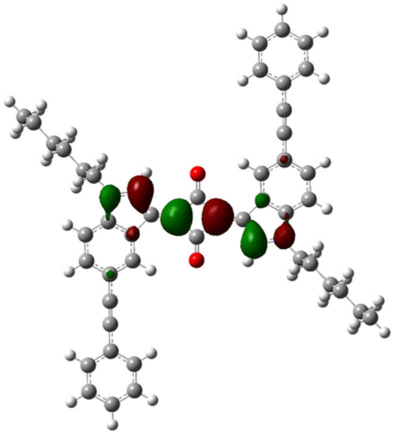

**ISD-6**	|1><br>w=1.00269<br>f=1.0217<br>2.2771eV	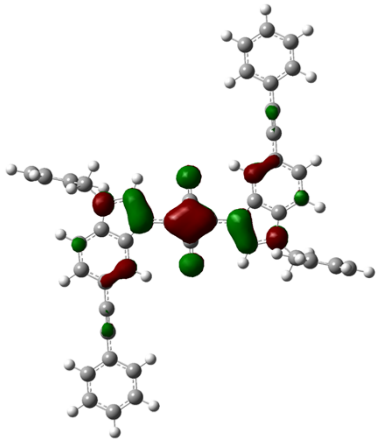	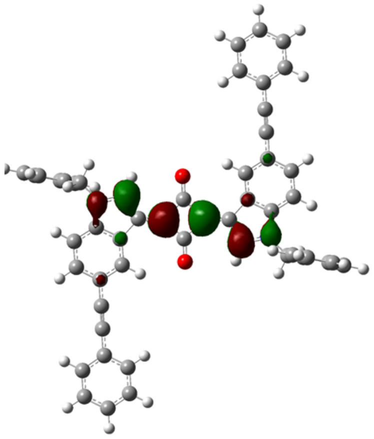
	|2><br>w=0.99543<br>f=0.0000<br>2.5996eV	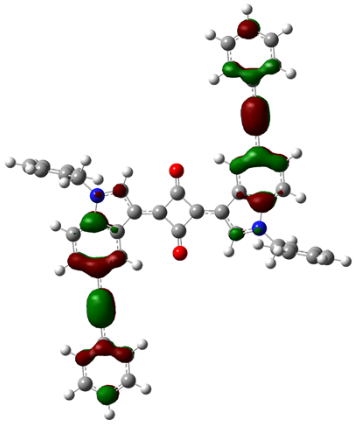	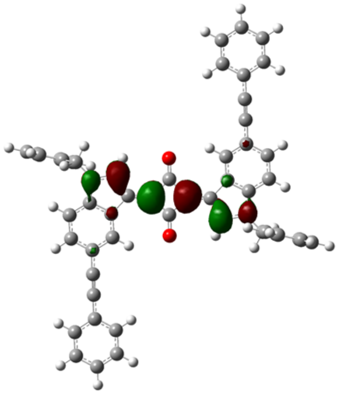
**ISD-7**	|1><br>w=1.01441<br>f=1.1726<br>2.3921eV	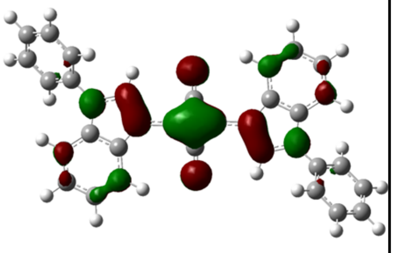	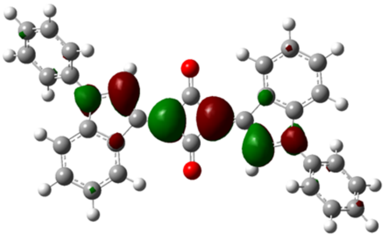
	|2><br>w=0.97702<br>f=0.0000<br>3.2494eV	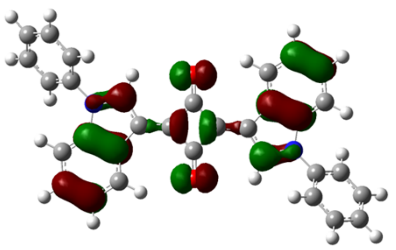	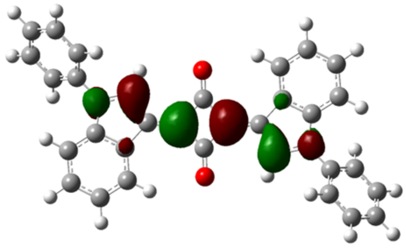

Fig.S1. Natural transition orbitals describing the first and second optimized ground states (absorption). The numbers in the left column indicate the corresponding excited-state number, fraction of the NTO pair contribution into the given electronic excitation, w; the excitation energy in eV; and f, the oscillator strength for the one-photon-absorption (OPA) excitations.

3. **Digital photo of ISDs in solution**

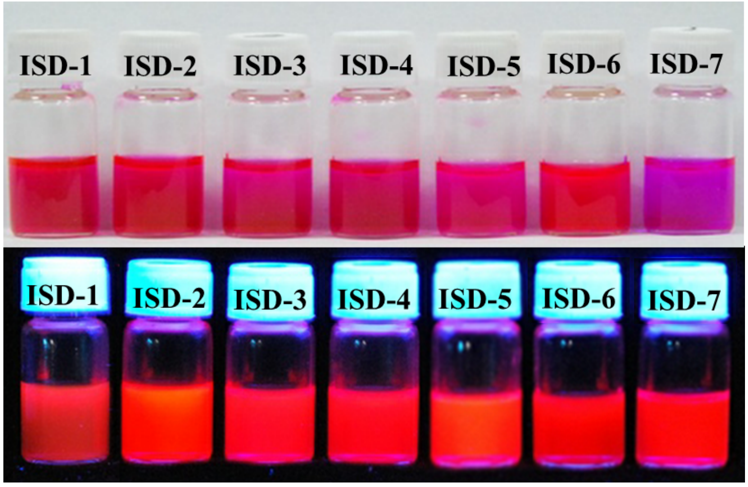

Fig.S2. Digital photo of ISDs in THF (top) and under UV irradiation (bottom).

4. **The time-resolved fluorescence spectra**

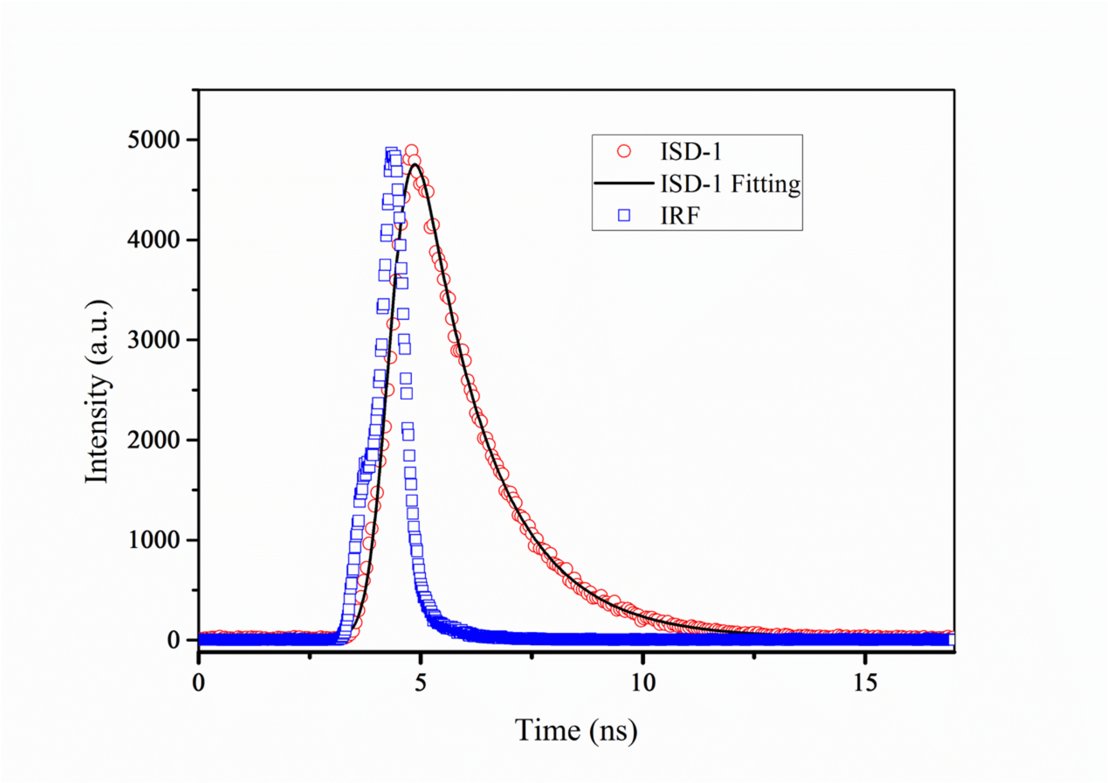

τ=1.58ns R^2=0.998

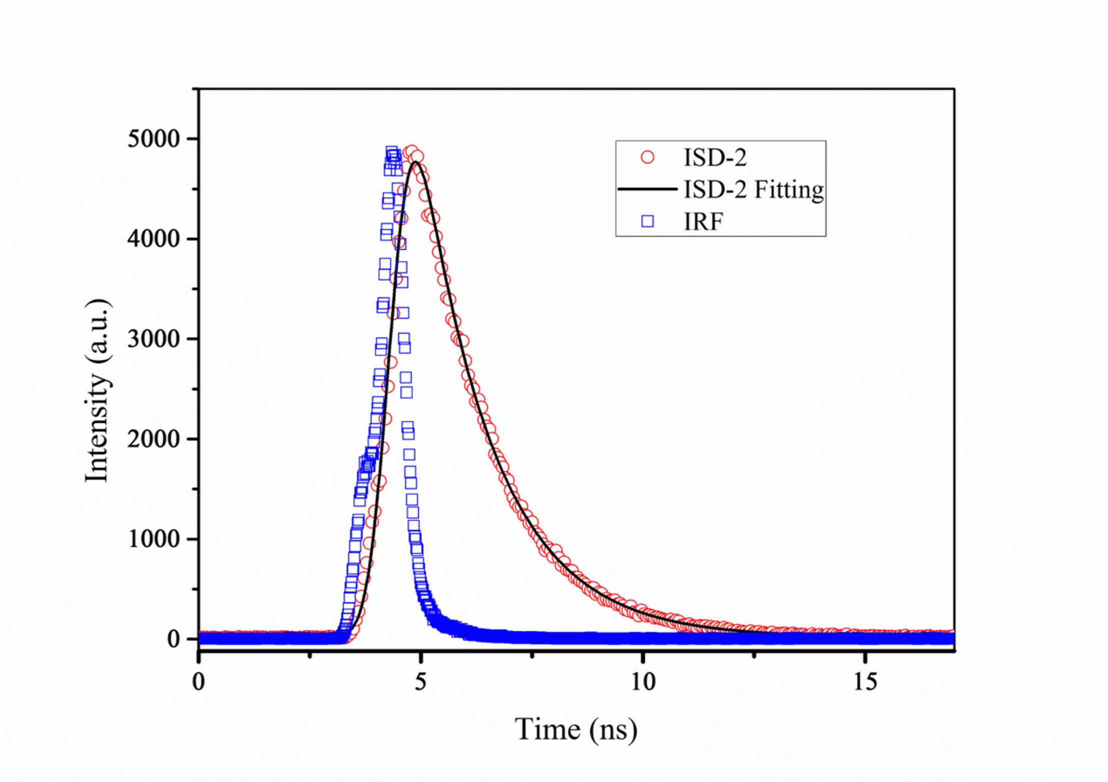

τ=1.65ns R^2=0.998

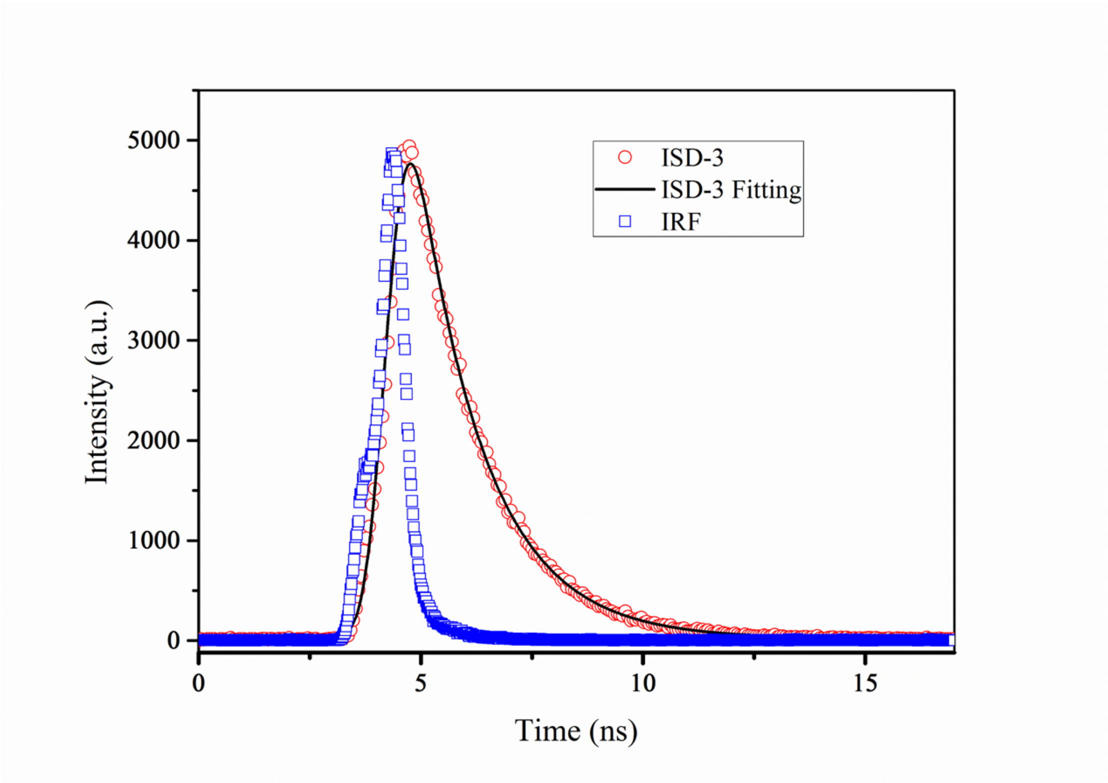

τ=1.53ns R^2=0.998

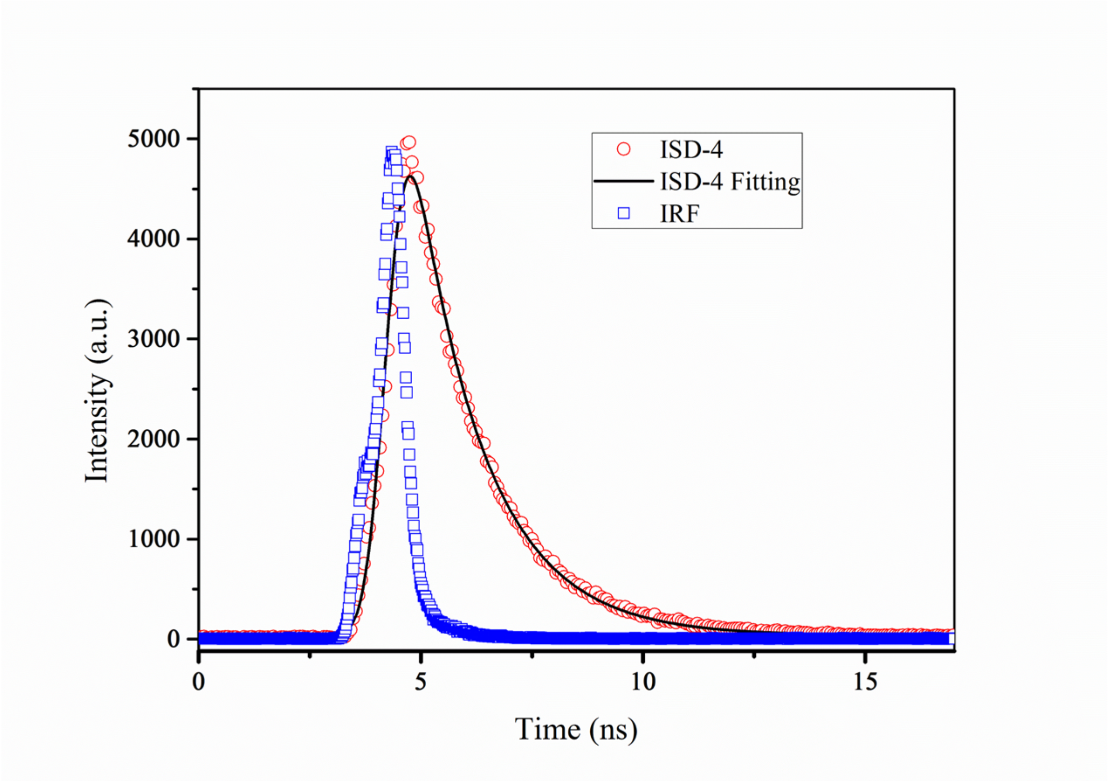

τ=1.57ns R^2=0.997

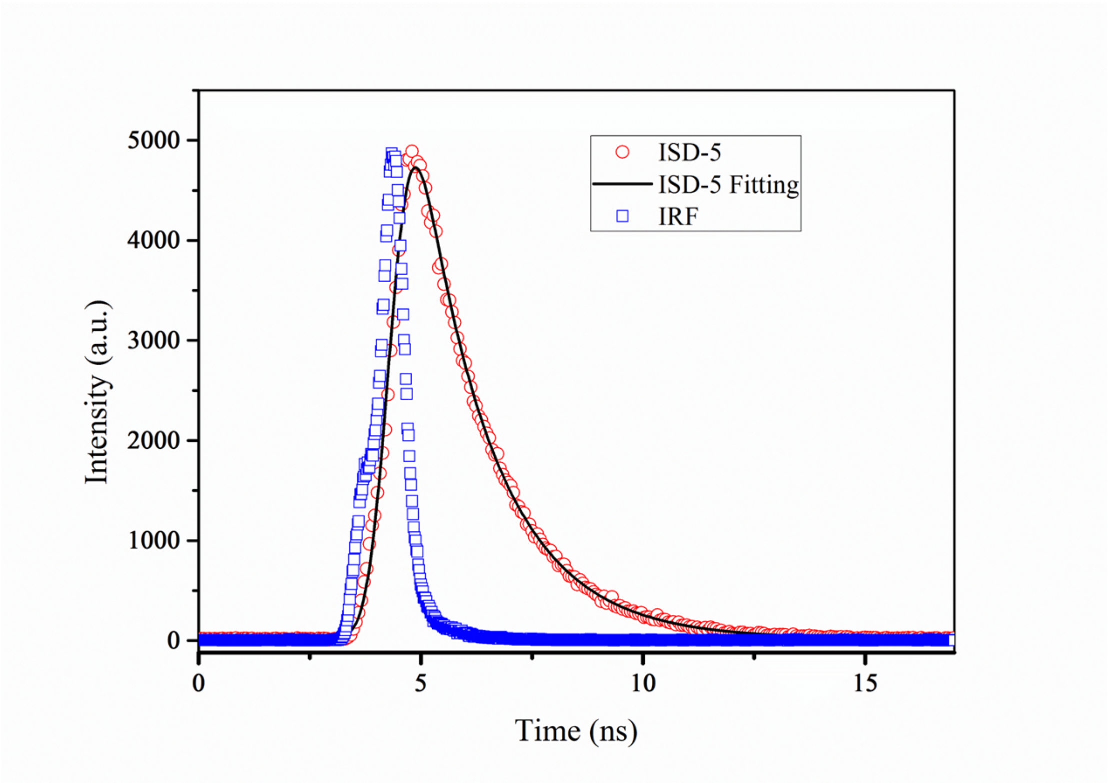

τ=1.64ns R^2=0.998

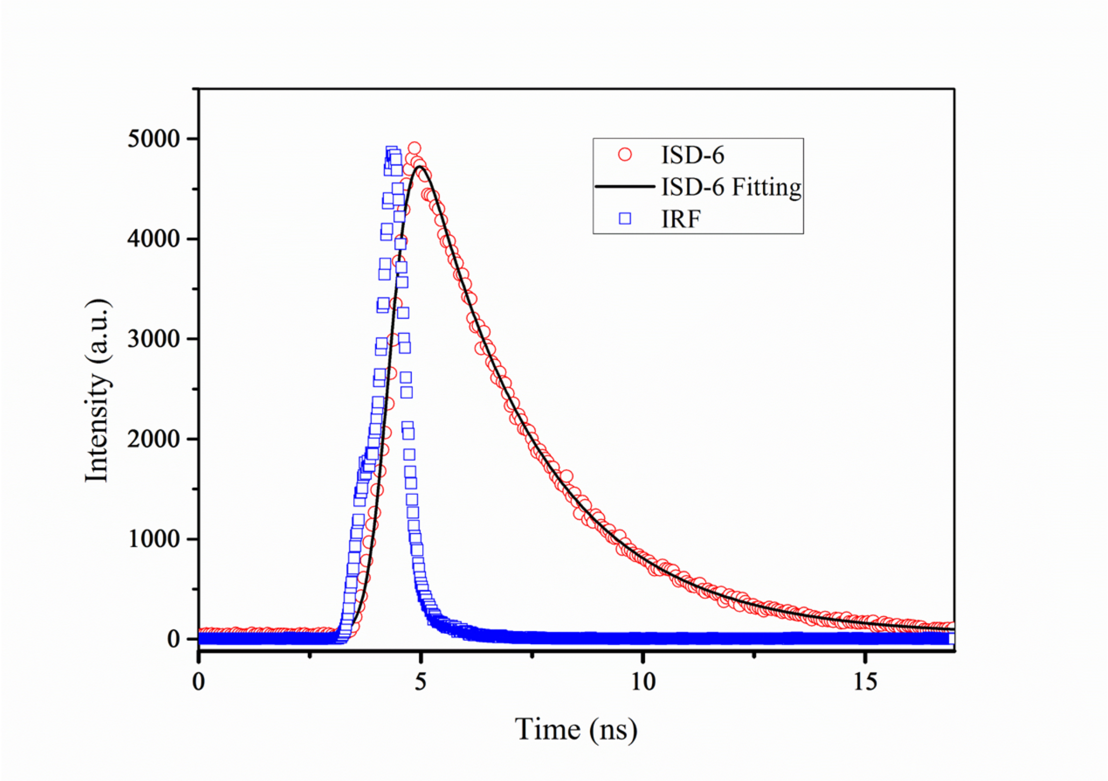

τ=1.67ns R^2=0.999

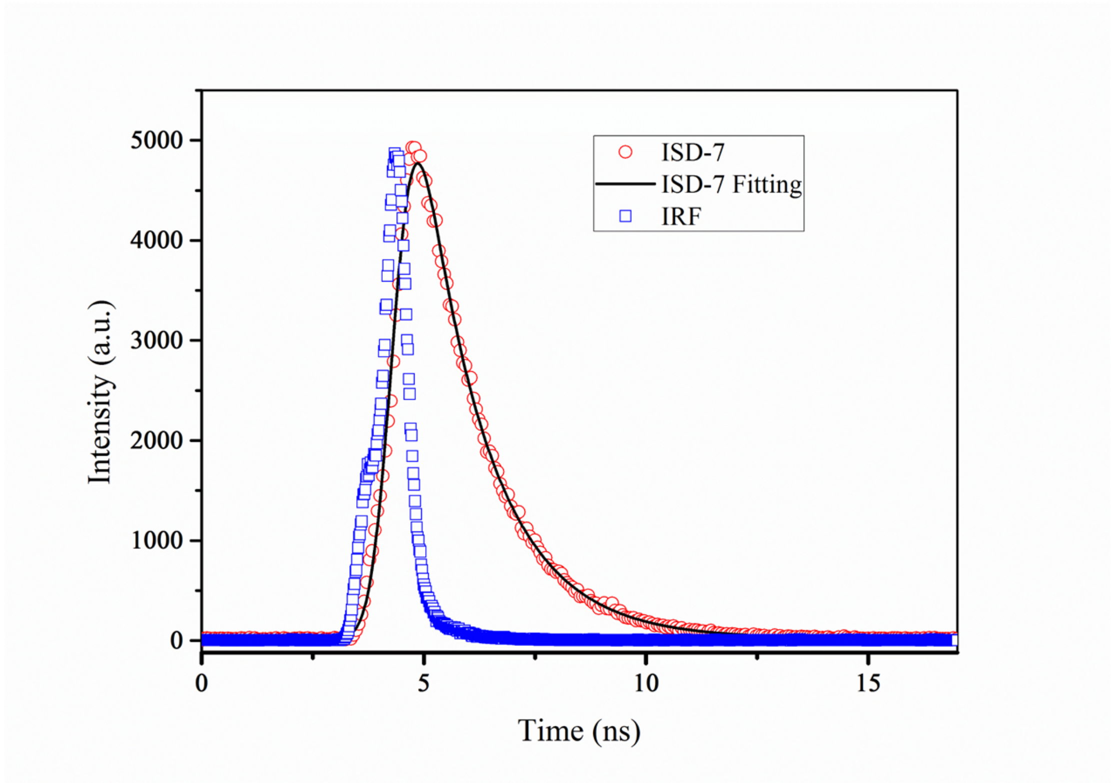

τ=1.47ns R^2=0.998

Fig.S3. The time-resolved fluorescence spectra of ISDs in THF solutions. The scatter dot present the experiment fluorescence decay curves, and the black curves show the fitting line. The instrument response function (IRF) was also show in the spectra. The life of fluorescence (τ) and coefficient of determination (R^2) are provided with each charts.

5. The cytotoxicity of the ISD-7 molecule

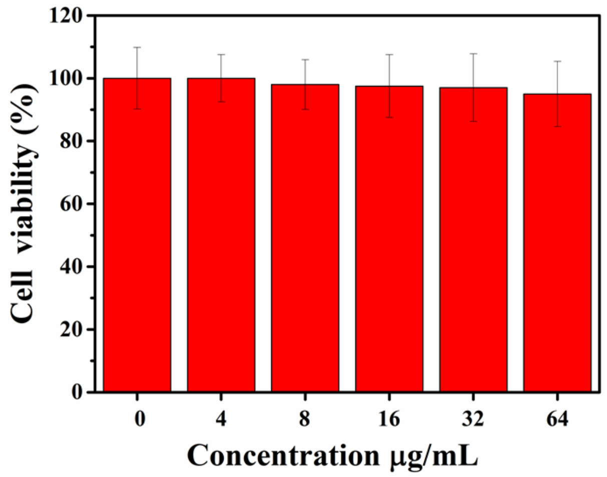

Fig. S4 Viability of MCF-7 cells at various concentrations of ISD-7.

To assess the cytotoxicity of the ISD-7 molecule, MCF-7 cells were grown in the presence of ISD-7 for 24 h, and the viability was observed after staining using Live/Dead kit. Cells were cultured in a 24-well microplate (approximately 10^5 cells per well). After culturing for 24 h, added

calcein and ethidium homodimer-1 PBS solution, and cultured for 30 min at normal culture environment. The cell viability was determined using an confocal microscope (Carl Zeiss LSM710). The cell viability was calculated by counting live (green) and dead (red) cells to that of the control well and expressed as a percentage. The viability of MCF-7 cells at various concentrations of ISD-7 was shown in Fig. S4.

6. Other ISD molecues studied theoretically

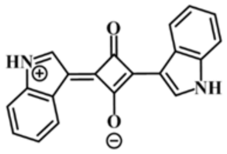

ISD-A

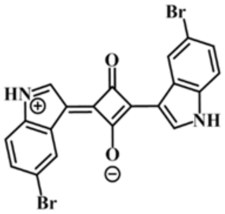

ISD-B

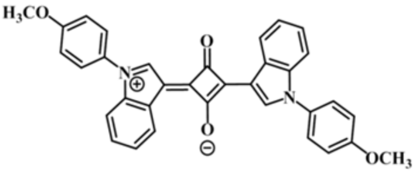

ISD-C

Molecules		HOLE	Particle
**ISD-A**	|1> W=1.02020 f= 0.8500 2.5395 eV	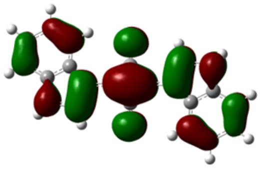	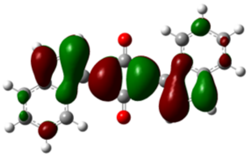
	|2> W=0.99809 f= 0.0000 3.2807 eV	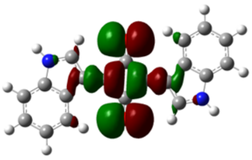	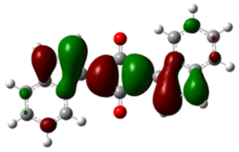
**ISD-B**	|1> W=1.01658 f= 0.8549 2.5005 eV	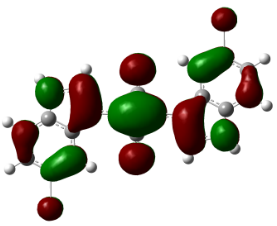	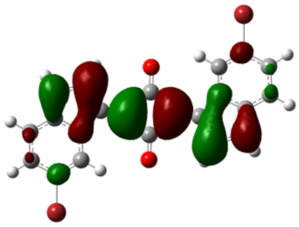
	|2> W=0.99972 f=0.0000 3.0361 eV	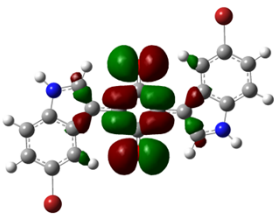	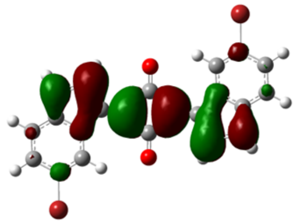
ISD-C	|1> w= 1.01320 f= 1.2492 2.3828 eV	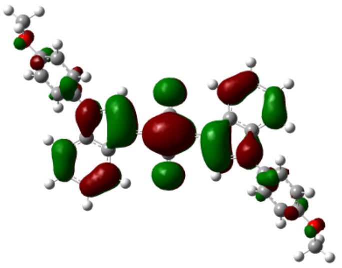	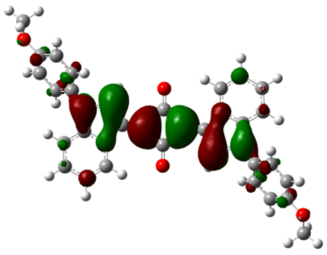

	|2><br>w= 0.98280<br>f=0.0000<br>3.2392eV	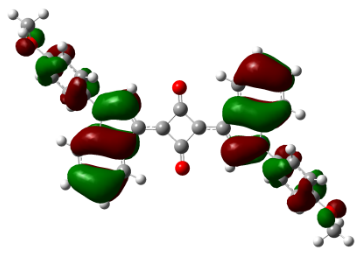	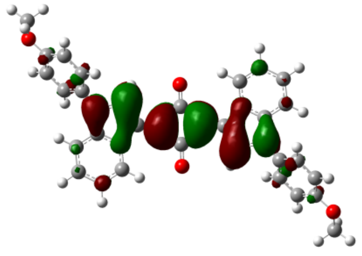

Fig. S5 The structures of other ISDs studied theoretically; and Natural transition orbitals describing the first and second optimized ground states (absorption). The numbers in the left column indicate the corresponding excited-state number, fraction of the NTO pair contribution into the given electronic excitation, w; the excitation energy in eV; and f, the oscillator strength for the one-photon-absorption (OPA) excitations.

Severally other ISDs have been studied theoretically, and three representative structures are shown in Fig. S5. The shapes of NTOs for ISD-A are similar with the counterparts with the alkyl chain i.e. ISD-1. Their final state excited electron delocalization is limited around the four-member ring. The structure of ISD-B can been considered as the ISD-2 with no-alkyl chain, however, the situations of NTOs is different with the ISD-2. The final state excited electron delocalization is also limited around the four-member ring. Although the structure of ISD-C is a small variation of of ISD-7, the hole of S_2 is separated by the four-member ring, which suggests that it may not be an ideal TPA candidate. The NTO calculation indicated that the excited states of ISD-A, ISD-B and ISD-C are localized in some part of the molecular structure, so they are unlikely to be exhibit good TPA properties, so that they were not synthesized. The calculation helped us to focus our investigation on a few structures, hence significantly reduced the synthetic work load.

7. Reference

1. S. Bahn, S. Imm, K. Mevius, L. Neubert, A. Tillack, J. M. Williams and M. Beller, *Chem. Eur. J.*, 2010, **16**, 3590-3593.
2. S. Potavathri, K. C. Pereira, S. I. Gorelsky, A. Pike, A. P. LeBris and B. DeBoef, *J. Am. Chem. Soc.*, 2010, **132**, 14676-14681.
3. Q. Li, J. Zou, J. Chen, Z. Liu, J. Qin, Z. Li and Y. Cao, *J. Phys. Chem. B*, 2009, **113**, 5816-5822.
4. D. A. Evans, K. R. Fandrick and H. J. Song, *J. Am. Chem. Soc.*, 2005, **127**, 8942-8943.
5. P. Y. Choy, C. P. Lau and F. Y. Kwong, *J. Org. Chem.*, 2011, **76**, 80-84.
6. L. Beverina, M. Crippa, P. Salice, R. Ruffo, C. Ferrante, I. Fortunati, R. Signorini, C. M. Mari, R. Bozio, A. Facchetti and G. A. Pagani, *Chem. Mater.*, 2008, **20**, 3242-3244.
7. P. Salice, J. Arnbjerg, B. W. Pedersen, R. Toftegaard, L. Beverina, G. a. Pagani and P. R. Ogilby, *J. Phys. Chem. A*, 2010, **114**, 2518-2525.
8. (a) X.-F. Zhang, Q. Xi and J. Zhao, *J. Mater. Chem.*, 2010, **20**, 6726; (b) M. Akhyar Farrukh, *Advanced Aspects of Spectroscopy*, InTech, 2012.
